# Coevolution between MHC Class I and Antigen-Processing Genes in Salamanders

**DOI:** 10.1093/molbev/msab237

**Published:** 2021-08-10

**Authors:** Gemma Palomar, Katarzyna Dudek, Magdalena Migalska, J W Arntzen, G Francesco Ficetola, Dušan Jelić, Elizabeth Jockusch, Inigo Martínez-Solano, Masatoshi Matsunami, H Bradley Shaffer, Judit Vörös, Bruce Waldman, Ben Wielstra, Wiesław Babik

**Affiliations:** 1 Institute of Environmental Sciences, Faculty of Biology, Jagiellonian University, Kraków, Poland; 2 Naturalis Biodiversity Center, Leiden, The Netherlands; 3 Institute of Biology, Leiden University, Leiden, The Netherlands; 4 Department of Environmental Sciences and Policy, University of Milan, Milan, Italy; 5 Laboratoire d’Ecologie Alpine (LECA), CNRS, Université Grenoble Alpes and Université Savoie Mont Blanc, Grenoble, France; 6 Croatian Institute for Biodiversity, Zagreb, Croatia; 7 Ecology and Evolutionary Biology, University of Connecticut, Storrs, CT, USA; 8 Museo Nacional de Ciencias Naturales (MNCN), Consejo Superior de Investigaciones Científicas (CSIC), Madrid, Spain; 9 Department of Advanced Genomic and Laboratory Medicine, Graduate School of Medicine, University of the Ryukyus, Nishihara-cho, Japan; 10 Department of Ecology and Evolutionary Biology, University of California, Los Angeles, Los Angeles, CA, USA; 11 La Kretz Center for California Conservation Science, Institute of the Environment and Sustainability, University of California, Los Angeles, Los Angeles, CA, USA; 12 Department of Zoology, Hungarian Natural History Museum, Budapest, Hungary; 13 Department of Integrative Biology, Oklahoma State University, Stillwater, OK, USA; 14 School of Biological Sciences, Seoul National University, Seoul, South Korea

**Keywords:** major histocompatibility complex, antigen-processing genes, coevolution, Urodela, comparative methods

## Abstract

Proteins encoded by antigen-processing genes (APGs) provide major histocompatibility complex (MHC) class I (MHC-I) with antigenic peptides. In mammals, polymorphic multigenic *MHC-I* family is served by monomorphic APGs, whereas in certain nonmammalian species both *MHC-I* and APGs are polymorphic and coevolve within stable haplotypes. Coevolution was suggested as an ancestral gnathostome feature, presumably enabling only a single highly expressed classical *MHC-I* gene. In this view coevolution, while optimizing some aspects of adaptive immunity, would also limit its flexibility by preventing the expansion of classical *MHC-I* into a multigene family. However, some nonmammalian taxa, such as salamanders, have multiple highly expressed *MHC-I* genes, suggesting either that coevolution is relaxed or that it does not prevent the establishment of multigene *MHC-I*. To distinguish between these two alternatives, we use salamanders (30 species from 16 genera representing six families) to test, within a comparative framework, a major prediction of the coevolution hypothesis: the positive correlation between *MHC-I* and APG diversity. We found that *MHC-I* diversity explained both within-individual and species-wide diversity of two APGs, *TAP1* and *TAP2*, supporting their coevolution with *MHC-I*, whereas no consistent effect was detected for the other three APGs (*PSMB8*, *PSMB9*, and *TAPBP*). Our results imply that although coevolution occurs in salamanders, it does not preclude the expansion of the *MHC-I* gene family. Contrary to the previous suggestions, nonmammalian vertebrates thus may be able to accommodate diverse selection pressures with flexibility granted by rapid expansion or contraction of the *MHC-I* family, while retaining the benefits of coevolution between *MHC-I* and *TAPs*.

## Introduction

Adaptive immunity is a major vertebrate innovation (Müller et al. 2018). The major histocompatibility complex (MHC) is a key player in the adaptive immunity of jawed vertebrates (Flajnik 2018). Classical MHC proteins present antigenic peptides to T cells, which, upon recognition of foreign antigens, trigger an adaptive immune response. Classical class I molecules (MHC-I) enable general surveillance of the translational activity inside cells, by presentation on the cell surface of antigens derived from intracellular proteins (including those of viruses and intracellular bacteria). The antigens are generated and loaded onto MHC-I molecules in a carefully orchestrated process (Blum et al. 2013; Blees et al. 2017; Fisette et al. 2020; Trowitzsch and Tampé 2020). First, antigenic peptides are produced by a dedicated version of the proteasome: immunoproteasome. The three immunoproteasome-specific catalytic units are encoded by *PSMB8* (*LMP7*), *PSMB9* (*LMP2*), and *PSMB10* (*MECL1*) genes (reviewed in Murata et al. [2018]). Next, the peptides produced by the immunoproteasome are pumped from the cytoplasm to the lumen of the endoplasmatic reticulum by a specialized, heterodimeric transporter associated with antigen presentation (TAP, encoded by the *TAP1* and *TAP2* genes). Peptides are then loaded onto MHC-I molecules with the help of several proteins, including tapasin (encoded by the *TAPBP* gene), a chaperone and mediator of TAP–MHC-I interaction. Tapasin stabilizes an empty MHC-I molecule and ensures loading of high-affinity peptides. The collective term antigen-processing genes (APGs) is used for the genes encoding PSMB, TAP, and TAPBP proteins (e.g., McConnell et al. 2016; Palomar et al. 2021). Finally, the antigen–MHC-I complex moves via a secretory pathway to the cell surface.

The above description applies to the classical *MHC-I* genes, which are highly expressed in multiple tissues, highly polymorphic, and encode proteins presenting antigenic peptides to the cytotoxic CD8+ αβ T cells. In addition, so-called nonclassical *MHC-I* genes, often, but not always linked to the classical *MHC-I* genes, have been identified in all vertebrates studied to date (Adams and Luoma 2013). They encode molecules similar in sequence and structure to the classical MHC-I, but of limited polymorphism and tissue expression. Their functions are less well defined, but often involve presentation of specialized antigen types, interaction with other cell types, such as NK or NKT cells (Adams and Luoma 2013; Edholm et al. 2013), and may or may not require input from APGs (Braud et al. 1998; Tilloy et al. 1999).

The dependence of classical MHC-I on ligands supplied by the products of APGs sets the stage for coevolution between APGs and *MHC-I* within species (Kaufman 1999). In its essence, the coevolution hypothesis posits that some combinations of APG and MHC-I alleles work better together than others because selection has optimized their interaction (Kaufman 1999, 2015). High polymorphism, maintained mainly by the arms-race between hosts and their pathogens, is a defining feature of both classes of classical MHC (Radwan et al. 2020). This polymorphism, concentrated in the peptide-binding groove of MHC molecules, affects the spectrum of antigens that can be bound. As APG products should supply antigenic peptides matching the requirements of MHC-I alleles, APG polymorphism is expected under coevolution as well. With both coevolving partners exhibiting high levels of polymorphism, an efficient system would require little or no recombination between them—frequent recombination would impose a heavy genetic load, separating coadapted alleles and thereby reducing the fitness of recombinant haplotypes (Kaufman 1999). Tight linkage between APG and *MHC-I* enables coevolution and is thus a key prediction of the hypothesis that has been confirmed in several vertebrate groups, such as frogs (Ohta et al. 2006), salamanders (Palomar et al. 2021), and birds (Kaufman et al. 1999). Mammals are a notable exception (Horton et al. 2004), where generalist APGs serve all MHC-I alleles, presumably after the linkage between *MHC-I* and APGs was broken by an inversion (Kaufman 2015). Tight linkage was proposed as the ancestral gnathostome condition (Ohta et al. 2006), leading to the hypothesis that APG–*MHC-I* coevolution is also an ancestral gnathostome feature (reviewed in Ohta and Flajnik [2015] and Kaufman [2018b]).

By one view, coevolutionary fine-tuning between the APGs and MHC-I should lead to a single highly expressed classical *MHC-I* gene, as observed, for example, in the chicken (Kaufman et al. 1999) and the frog *Xenopus* (Ohta et al. 2006), whereas the relaxation of coevolution would allow the appearance of a multigenic classical *MHC-I*, as seen in mammals (Kaufman 2018b). In this view, coevolution may be considered as an ancestral state that limits the flexibility of the adaptive immune response by restricting the number of classical *MHC-I* genes. However, evidence for multiple expressed *MHC-I* genes has accumulated in nonmammalian vertebrates: fishes (Nonaka and Nonaka 2010; McConnell et al. 2014; Grimholt et al. 2015), amphibians (Sammut et al. 1999; Fijarczyk et al. 2018; Palomar et al. 2021), and birds (Drews and Westerdahl 2019). At least some species in these groups maintain tight linkage between *MHC-I* and APGs (McConnell et al. 2016; Palomar et al. 2021). This suggests that either coevolution was relaxed in these species, or coevolution does not preclude establishment, following gene duplication, of multiple highly expressed *MHC-I* genes. Indeed, already in the early days of the coevolution hypothesis, it was postulated that multiple genes with similar peptide-binding specificities could coevolve on a haplotype with a single set of APG alleles (Kaufman 1999). An accumulation of *MHC-I* diversity in a population would then be coupled with an increase in the overall APG diversity, whereas monomorphic “average best fit” APGs would be expected to evolve in the absence of coevolution.

To date, most of what we know about coevolution between *MHC-I* and APGs comes from experimental studies in model systems with simple MHC, such as the chicken (Walker et al. 2011; van Hateren et al. 2013; Kaufman 2015). Such studies are invaluable in providing a detailed mechanistic understanding of the coevolutionary process, but few systems are amenable to full scale immunological and immunogenetic analyses. The question of whether APG–*MHC-I* coevolution is a widespread phenomenon should be addressed with studies over broader phylogenetic scales because the key predictions of the hypothesis can be tested within a comparative framework. Crucially, the coevolution hypothesis predicts a positive correlation between intraspecific APG and *MHC-I* diversity when differences in background genetic diversity are controlled. However, this prediction has not been tested so far, and, whereas *MHC-I* polymorphism has been studied in dozens of species from all major vertebrate groups, information on APG polymorphism is scarce outside mammals (Ohta et al. 2003; Miura et al. 2010; Walker et al. 2011; Huang et al. 2013; van Hateren et al. 2013; Fijarczyk et al. 2018).

Salamanders (Urodela) are characterized by a long, independent history ― they diverged from other extant amphibian lineages approximately 250–300 Ma, with a crown age estimated at approximately 180–200 Ma, (Marjanović and Laurin 2014; Irisarri et al. 2017; Jetz and Pyron 2018). Salamanders exhibit a combination of features that makes them a suitable model to test the coevolution hypothesis. At least some APGs are polymorphic in certain species (Huang et al. 2013; Fijarczyk et al. 2018; Palomar et al. 2021), as expected under the coevolution model. On the other hand, salamanders studied so far have multiple highly expressed *MHC-I* genes (Sammut et al. 1999; Fijarczyk et al. 2018; Palomar et al. 2021). This stands in contrast to predictions of the coevolution model which, as currently formulated, predicts a single, highly expressed classical *MHC-I* gene. We note, however, that establishing the classical nature of the *MHC-I* by sequence analysis alone is challenging (Sammut et al. 1999), whereas functional data are lacking in salamanders. To date, the only functional studies of nonclassical *MHC-I* in amphibians were conducted in *Xenopus* (Edholm et al. 2013; Robert and Edholm 2014; Banach et al. 2017), but *Xenopus* nonclassical class I genes appear to lack orthologues in salamanders (Sammut et al. 1999; Edholm et al. 2014).

Recently, we used salamanders to test two predictions of the coevolution hypothesis: 1) tight linkage between APGs and *MHC-I*, and 2) a signal of adaptive evolution in APGs (Palomar et al. 2021). First, we directly estimated the recombination rate between APG and *MHC-I* in *Lissotriton* newts by examining products of over 1,500 meioses. No recombination was detected between *MHC-I* and four of the five analyzed APGs, whereas the total map length of the region spanning multiple *MHC-I* genes and all five APGs was less than 0.5 cM. The extremely limited recombination between *MHC-I* and APGs in *Lissotriton* and close physical proximity of these genes revealed by the recent chromosomal-scale assembly of the axolotl genome (Schloissnig et al. 2021) suggest that salamanders fulfil a condition for coevolution. Second, we used the coding sequences of APGs derived from transcriptomes of over 40 salamander species to test for signatures of positive selection over evolutionary timescale. The signal of adaptive evolution was subtle and restricted mostly to *TAP1* and *TAP2* genes. We concluded that coevolution between APGs and *MHC-I* cannot be ruled out, but it may involve only some APGs, in particular *TAPs*, and its mechanisms would need to accommodate *MHC-I* duplication. We proposed that a major prediction of the coevolution hypothesis—a positive correlation between genetic variation of APGs and *MHC-I—*should be tested in a comparative framework. Here, we perform such an analysis.

**Fig. 1.**
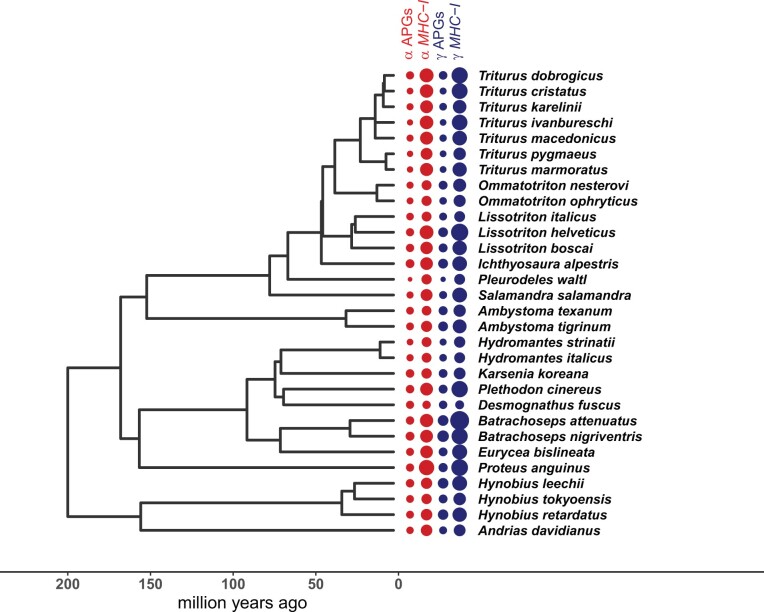
Phylogeny and diversity of *MHC-I* and APGs. Circle sizes are proportional to *MHC-I* and APG α and γ diversities, calculated for the sample sizes of 15 individuals per species (see Text). To facilitate graphical presentation and an overall visual assessment, the scale of *MHC-I* diversities is 0.2× the scale of the APG diversities.

To test for the correlation between APG and *MHC-I* diversity, we examined 30 salamander species widely sampled from the Urodela tree of life (fig. 1). Our sampling included representatives of six out of nine currently recognized salamander families (Frost 2021), and spans the most recent ancestor of all living species. We used this data set to assess the diversity of: 1) *MHC-I* genes, 2) five APGs (*PSMB8*, *PSMB9*, *TAP1*, *TAP2*, and *TAPBP*), and 3) five non-APGs (*BRD2*, *DAXX*, *KIFC1*, *RXRBA*, *RGL2*)—protein coding genes that are physically (Palomar et al. 2021), but not functionally, tightly linked to *MHC-I*. Sequence polymorphism was measured both at synonymous codon positions and at the amino acid sequence level. Diversity was estimated both at the individual and at the species level, using measures applicable to all examined genes. These measures, adopted from biodiversity studies (Chao et al. 2014), allow a comprehensive characterization of diversity, taking into account sequence divergence between alleles as well as differences in copy number among genes and individuals. We then fitted several phylogenetic generalized least squares (PGLS) models to the data, to test whether APG diversity could be explained by *MHC-I* and non-APG diversity, as predicted by the coevolution hypothesis.

## Results

### Sequencing and Polymorphism

#### Samples

Diversity of *MHC-I*, APGs, and non-APGs was studied, by targeted sequencing of genomic DNA, in 30 species representing 16 genera (23% of salamander genera), and six out of nine Urodela families that comprise approximately 98% salamander species (fig. 1). One to four (median = 2) populations and 15–65 (median = 35) individuals per species were examined (supplementary table S1, Supplementary Material online).

#### MHC-I

**Fig. 2.**
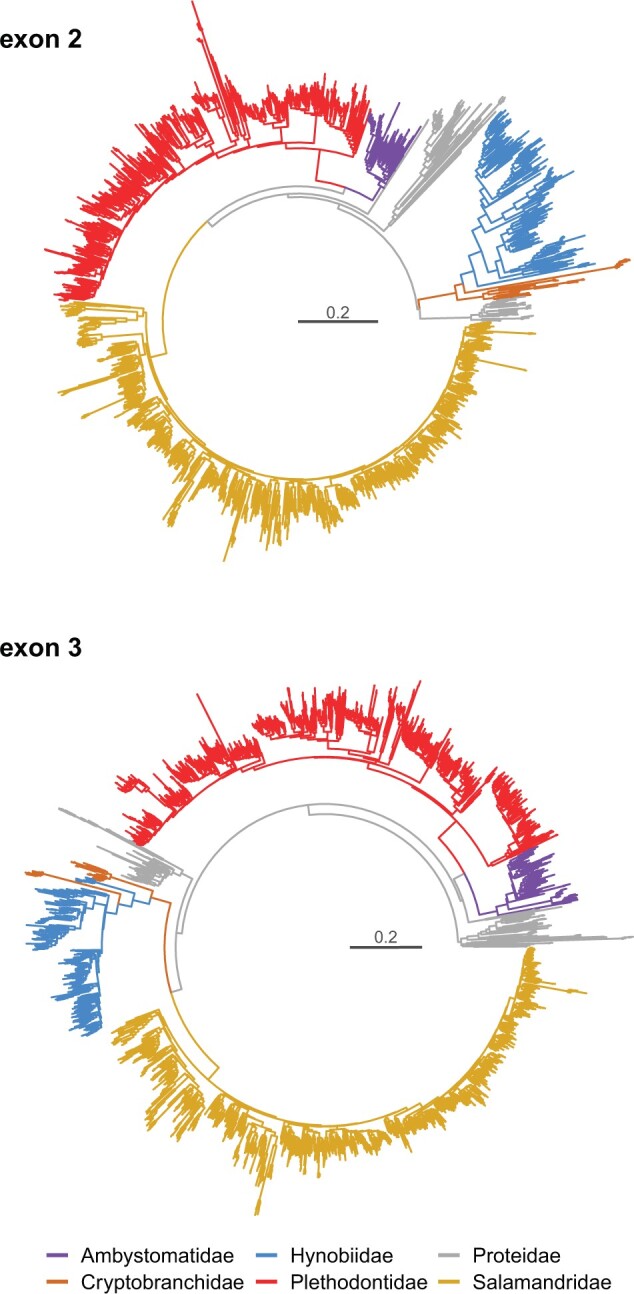
Phylogenies of salamander *MHC-I* alleles. BIONJ trees show relationships for putative functional exon 2 and exon 3 alleles. The trees were constructed from matrices of Jukes–Cantor distances and color-coded according to salamander families.

Polymorphism was assessed using Illumina amplicon sequencing for 214–224 bp of exon 2 and 166–184 bp of exon 3, depending on genus and not counting indels that caused some sequences to depart from the canonical length. Full codon data available for all species covered amino acid positions 8–79 (exon 2) and 109–162 (exon 3) of the human HLA-A protein α domain. A total of 2,796 and 3,133 unique sequence variants were detected in exon 2 and exon 3, respectively (supplementary fig. S1, Supplementary Material online). Although we cannot assign these variants to loci, for simplicity, we will refer to them as “alleles.” Nonetheless, the alleles come from multiple genes as individuals typically carry more than two *MHC-I* variants. Genotyping repeatability, averaged over all species, was 91.4% for exon 2 and 97.4% for exon 3 (supplementary table S2, Supplementary Material online). The fraction of nonfunctional alleles (exhibiting frameshifts or internal stop codons) was generally low (on an average 5.6% and 4.4% for exons 2 and 3, respectively), but with considerable variation among species (supplementary table S3, Supplementary Material online). The mean per-individual number of potentially functional alleles ranged from 2.6 (*Desmognathus fuscus*, Plethodontidae) to 21.1 (*Lissotriton helveticus*, Salamandridae) in exon 2, and from 1.8 (*D. fuscus*) to 31.3 (*Proteus anguinus*, Proteidae) in exon 3, indicating substantial differences in the number of *MHC-I* genes among salamander species (supplementary table S3, Supplementary Material online). The assayed fragments of exons 2 and 3 covered, respectively, one and four residues which are important for anchoring the termini of antigenic peptides and are conserved in classical MHC-I of most taxa. The alleles preserving the conserved amino acids at these residues formed the “conserved anchor” data set. This data set was intended to minimize the fraction of nonclassical alleles, but, because of the difficulties of establishing the classical/nonclassical status based on sequence alone, it may include some nonclassical alleles and exclude some classical alleles; therefore, we adopt a neutral “conserved anchor” name. The fraction of conserved anchor alleles in exon 2 ranged from 0.85 (*Andrias davidianus*, Cryptobranchidae) to 1.0 (*Desmognathus*, *Karsenia*, and *Salamandra*) and in exon 3, it ranged from 0.36 (*P. anguinus*) to 1.0 (*D. fuscus*) (supplementary table S3, Supplementary Material online). Phylogenies showed family-level monophyly of *MHC-I* alleles in most cases (fig. 2)—either gene duplications postdated the divergence of salamander families, or sequences of different genes have been homogenized in a process of concerted evolution. Whichever the mechanism, phylogenies show dynamic evolution of *MHC-I* in salamanders, making identification of 1:1 orthologs between families next to impossible. A detailed analysis of *MHC-I* molecular evolution in salamanders will be reported elsewhere (Minias et al., in preparation).

#### Antigen-Processing Genes

**Fig. 3.**
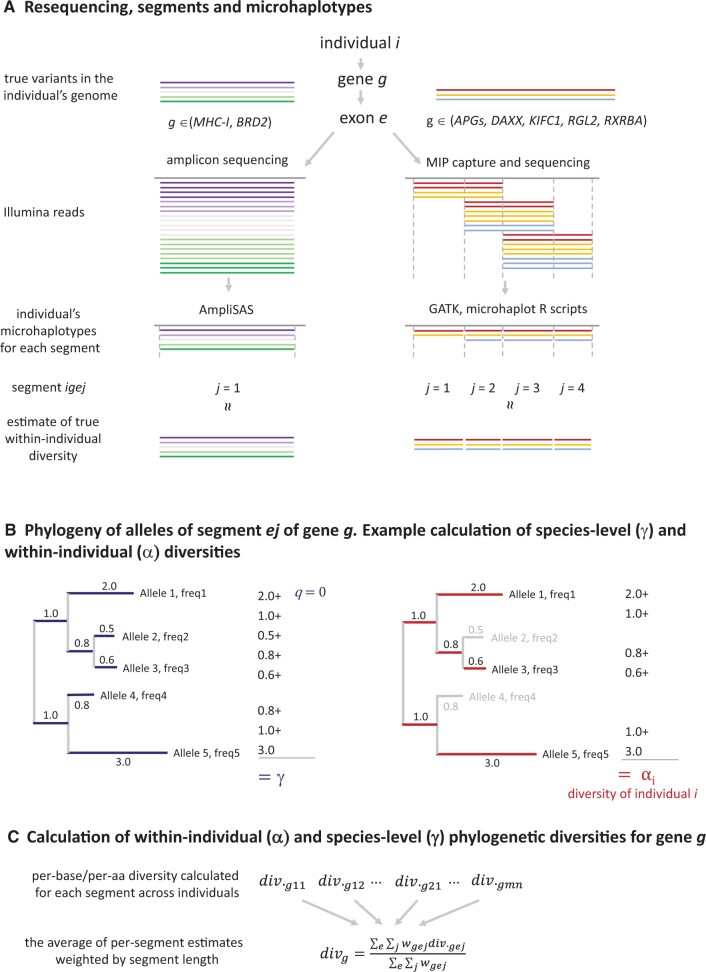
Sequencing and diversity estimation. (*A*) Resequencing of our genes of interest produced stacks of overlapping reads that provided physically phased microhaplotypes (local haplotypes). Amplicon sequencing (left) produced a single stack of reads per exon, whereas MIP resequencing (right) produced several, partly overlapping stacks, which were then divided into segments, such that within each segment microhaplotypes spanning the full segment length were recovered from reads spanning the segment. Thus a single segment per exon was obtained from amplicon resequencing, whereas MIP resequencing typically produced multiple segments per exon. Note that in both illustrated cases the individual has more than two alleles per gene, indicating gene duplication. In addition, not all variation is necessarily recovered using the applied methods, as indicated by one haplotype missed by MIP resequencing for segment *j *=* *1. (*B*) Phylogenetic γ (left) and α (right) diversities are then calculated for each segment, using the segment’s phylogeny, to estimate species-wide and individual-level diversity, respectively; γ diversity is the sum of branch lengths in the phylogeny of all alleles detected in a species (blue), with allele frequency weighting schemes depending on the *q* value (e.g., equal weights when *q *=* *0, see Text for details on *q *=* *1 and *q *=* *2); α diversity is the sum of branch lengths connecting alleles present in a given individual (red). (*C*) To calculate the per-gene α and γ phylogenetic diversities, the per-base/amino acid estimates were obtained using the method of Chao et al. (2014) and then their weighted average was calculated with segment length used as weights (see Text for details).

Polymorphism of all five APGs was assessed with Illumina sequencing of targets captured with molecular inversion probes (MIPs, fig. 3). Because sequencing produced stacks of overlapping paired-end reads starting at defined positions, we were able to obtain physically phased microhaplotypes for nonoverlapping segments along the reference (fig. 3). Apparently, some APGs have been lost in plethodontid salamanders: *PSMB8* was not found in the transcriptome of any plethodontid, *PSMB9* was not found in *Hydromantes* and *TAP2* may be missing in *Karsenia* (Palomar et al. 2021). We attempted to sequence as much APGs coding sequence (cds) as possible, but we were not able to design MIPs for exons shorter than approximately 120 bp. We considered a segment within an individual effectively sequenced if at least one MIP spanning that segment had a coverage of 20 or more reads. The average fraction of APG cds length effectively sequenced in at least 50% of individuals within species was 0.626 (4,289 bp), ranging from 0.528 (2,192 bp) in *Karsenia koreana* to 0.715 (5,116 bp) in *L. italicus* (supplementary table S4, Supplementary Material online). Detailed sequencing statistics are provided in supplementary tables S4–S6, Supplementary Material online, summary of TAP1 and TAP2 polymorphism in key residues is in supplementary table S7, Supplementary Material online and diversity estimates are in table 1 and supplementary table S8, Supplementary Material online.

**Table 1.** Summary of Diversities of *MHC-I (MHC)*, APGs (APG), and Non-APGs (nAPG) for All Species.

			Within-Individual (α, *q *=* *0) Diversity									Species-Wide (γ, *q *=* *1) Diversity								
			AA			AAGhm			d*S*			AA			AAGhm			d*S*		
Family	Genus	Species	APG	*MHC*	nAPG	APG	*MHC*	nAPG	APG	*MHC*	nAPG	APG	*MHC*	nAPG	APG	*MHC*	nAPG	APG	*MHC*	nAPG
Ambystomatidae	*Ambystoma*	*texanum*	0.036	0.457	0.012	0.021	0.407	0.007	0.072	0.438	0.023	0.065	0.936	0.018	0.043	0.837	0.011	0.131	0.788	0.039
Ambystomatidae	*Ambystoma*	*tigrinum*	0.037	0.819	0.008	0.027	0.725	0.005	0.091	0.793	0.018	0.088	1.426	0.009	0.066	1.183	0.006	0.178	1.032	0.027
Cryptobranchidae	*Andrias*	*davidianus*	0.029	1.099	0.003	0.019	0.939	0.003	0.051	1.919	0.010	0.038	0.842	0.003	0.025	0.810	0.003	0.062	1.693	0.012
Plethodontidae	*Batrachoseps*	*attenuatus*	0.050	1.554	0.007	0.032	1.378	0.005	0.104	1.342	0.027	0.121	3.649	0.010	0.084	3.069	0.007	0.227	1.932	0.039
Plethodontidae	*Batrachoseps*	*nigriventris*	0.063	1.451	0.009	0.041	1.269	0.008	0.132	1.420	0.028	0.156	2.399	0.014	0.110	2.129	0.012	0.278	1.995	0.045
Plethodontidae	*Desmognathus*	*fuscus*	0.044	0.245	0.004	0.024	0.210	0.002	0.079	0.151	0.008	0.050	0.311	0.004	0.029	0.255	0.002	0.099	0.187	0.008
Plethodontidae	*Eurycea*	*bislineata*	0.041	1.295	0.006	0.024	1.161	0.004	0.107	1.098	0.016	0.060	1.996	0.008	0.037	1.602	0.004	0.161	1.293	0.021
Plethodontidae	*Hydromantes*	*italicus*	0.025	0.539	0.008	0.020	0.491	0.006	0.040	0.322	0.007	0.032	0.593	0.009	0.025	0.549	0.006	0.045	0.362	0.007
Plethodontidae	*Hydromantes*	*strinatii*	0.018	0.554	0.006	0.014	0.510	0.004	0.031	0.347	0.008	0.022	0.733	0.008	0.016	0.631	0.006	0.041	0.389	0.009
Hynobiidae	*Hynobius*	*leechii*	0.052	0.903	0.014	0.032	0.787	0.008	0.115	1.177	0.059	0.117	2.149	0.015	0.080	1.630	0.009	0.222	2.142	0.085
Hynobiidae	*Hynobius*	*retardatus*	0.038	1.208	0.011	0.027	1.107	0.006	0.125	1.467	0.029	0.111	2.080	0.015	0.080	1.892	0.009	0.188	1.809	0.062
Hynobiidae	*Hynobius*	*tokyoensis*	0.037	0.637	0.020	0.023	0.568	0.016	0.102	0.796	0.051	0.060	1.121	0.026	0.037	0.902	0.021	0.167	1.347	0.078
Salamandridae	*Ichthyosaura*	*alpestris*	0.062	1.039	0.010	0.037	0.863	0.007	0.149	1.090	0.024	0.090	1.494	0.016	0.053	1.388	0.011	0.226	1.329	0.042
Plethodontidae	*Karsenia*	*koreana*	0.054	0.735	0.003	0.041	0.680	0.003	0.075	0.474	0.011	0.058	0.954	0.003	0.045	1.041	0.004	0.092	0.573	0.013
Salamandridae	*Lissotriton*	*boscai*	0.047	1.236	0.005	0.029	1.043	0.003	0.107	1.530	0.017	0.062	1.845	0.010	0.040	1.413	0.006	0.146	1.489	0.022
Salamandridae	*Lissotriton*	*helveticus*	0.045	1.612	0.021	0.024	1.364	0.015	0.144	1.724	0.033	0.086	2.924	0.038	0.050	2.267	0.024	0.237	1.739	0.061
Salamandridae	*Lissotriton*	*italicus*	0.033	0.506	0.017	0.024	0.422	0.009	0.071	0.512	0.033	0.038	0.654	0.019	0.027	0.523	0.010	0.102	0.584	0.039
Salamandridae	*Ommatotriton*	*nesterovi*	0.025	0.547	0.013	0.017	0.461	0.010	0.062	0.693	0.043	0.061	0.985	0.018	0.041	0.845	0.014	0.144	1.046	0.071
Salamandridae	*Ommatotriton*	*ophryticus*	0.021	0.561	0.015	0.012	0.457	0.010	0.050	0.652	0.048	0.031	0.972	0.017	0.018	0.719	0.011	0.105	0.931	0.055
Plethodontidae	*Plethodon*	*cinereus*	0.051	1.491	0.022	0.032	1.333	0.015	0.088	1.190	0.048	0.062	2.303	0.029	0.040	2.173	0.022	0.126	1.181	0.065
Salamandridae	*Pleurodeles*	*waltl*	0.005	0.599	0.003	0.002	0.532	0.003	0.005	0.820	0.003	0.006	0.699	0.003	0.003	0.554	0.004	0.006	0.810	0.004
Proteidae	*Proteus*	*anguinus*	0.044	3.205	0.009	0.029	2.791	0.007	0.095	6.067	0.008	0.069	2.168	0.017	0.046	1.759	0.015	0.154	3.511	0.015
Salamandridae	*Salamandra*	*salamandra*	0.025	1.068	0.011	0.015	0.972	0.008	0.062	0.835	0.022	0.040	1.917	0.016	0.025	1.648	0.010	0.093	0.967	0.041
Salamandridae	*Triturus*	*cristatus*	0.018	1.684	0.003	0.008	1.460	0.002	0.029	2.059	0.009	0.021	2.171	0.004	0.010	1.989	0.003	0.039	2.240	0.010
Salamandridae	*Triturus*	*dobrogicus*	0.043	1.796	0.004	0.025	1.594	0.003	0.143	2.009	0.012	0.054	2.513	0.005	0.032	2.182	0.003	0.180	1.935	0.014
Salamandridae	*Triturus*	*ivanbureschi*	0.012	1.702	0.003	0.008	1.554	0.002	0.027	1.899	0.011	0.017	2.059	0.004	0.013	1.712	0.002	0.037	1.734	0.011
Salamandridae	*Triturus*	*karelinii*	0.023	1.580	0.015	0.015	1.436	0.010	0.033	1.827	0.019	0.034	1.362	0.019	0.022	1.253	0.013	0.054	1.595	0.035
Salamandridae	*Triturus*	*macedonicus*	0.023	1.403	0.006	0.015	1.240	0.006	0.030	1.605	0.016	0.027	1.547	0.006	0.018	1.375	0.006	0.031	1.437	0.016
Salamandridae	*Triturus*	*marmoratus*	0.014	1.486	0.005	0.010	1.326	0.004	0.028	1.799	0.008	0.019	1.258	0.006	0.013	1.309	0.004	0.036	1.635	0.024
Salamandridae	*Triturus*	*pygmaeus*	0.013	1.333	0.001	0.007	1.187	0.000	0.035	1.704	0.007	0.018	1.132	0.001	0.010	0.922	0.000	0.044	1.651	0.008

Note.—Diversities for individual genes and other *q* values are in supplementary table S8, Supplementary Material online. AA, amino acid *p*-distance; AAGhm, amino acid Grantham distance; d*S*, DNA divergence at synonymous sites.

#### Non-APGs

Polymorphism of four non-APGs was assessed with MIPs, whereas the fifth, *BRD2*, was amplified and sequenced similarly to *MHC-I*. Because coding sequences of some non-APGs are long, we did not attempt to maximize the fraction of non-APGs cds sequenced, but instead aimed to obtain enough data for a meaningful comparison with APGs. The average total length of sequenced non-APGs cds was 3,558 bp, ranging from 2,246 in *Ommatotriton nesterovi* and *O. ophryticus* to 4,174 bp in *L. italicus*. Detailed sequencing statistics are in supplementary tables S4–S6, Supplementary Material online and diversity estimates are provided in table 1 and supplementary table S8, Supplementary Material online.

### Diversity and the Phylogenetic Correlation between *MHC-I* and APGs

Diversity of each gene was estimated at both the within-individual (α diversity) and species-wide (γ diversity) level using three measures of genetic distance: 1) DNA divergence at synonymous sites, 2) protein divergence measured as amino acid *p*-distance, and 3) functional protein divergence measured as Grantham (1974) distance. Within-individual diversity was expressed as the sum of branch lengths of the phylogenetic tree linking individual’s alleles—in the case of two alleles this amounted to the genetic distance between them. Species-wide diversity was expressed as the sum of branch lengths of the tree linking all alleles detected in a species, with three different schemes of weighting the allele frequency (fig. 3, see Materials and Methods for details).

**Fig. 4.**
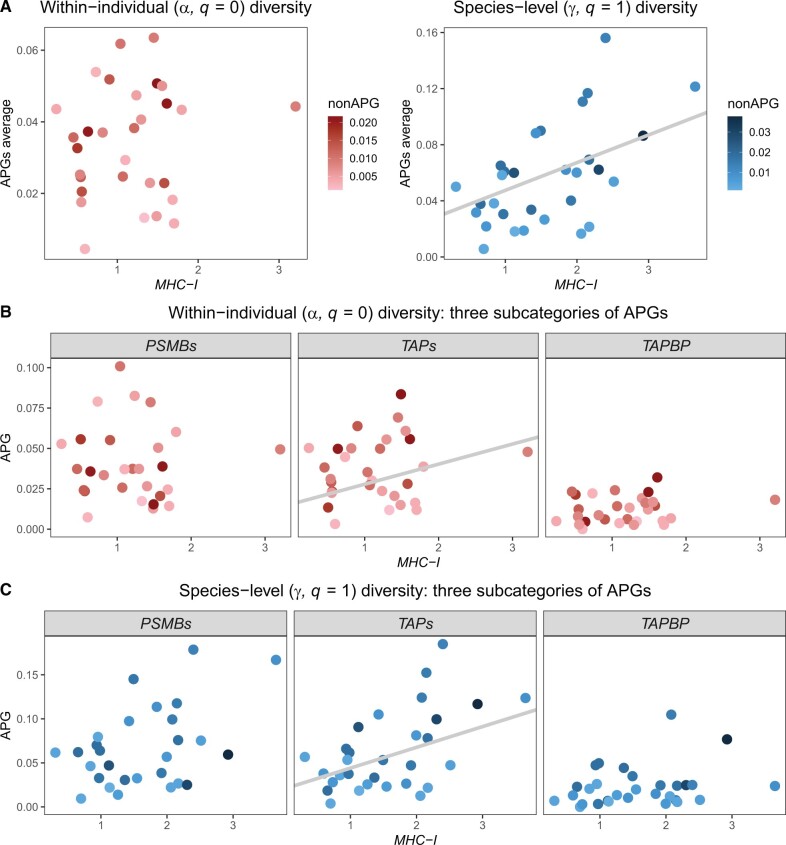
Relationship between APGs and *MHC-I* diversity. All plots show within-individual (α) and species-wide (γ) diversities, calculated using the proportion of different amino acids as a measure of genetic distance. For within individual diversity all variants were weighted equally (*q *=* *0), whereas for species-wide diversity, variants were weighted by their frequencies (*q *=* *1). The results were qualitatively similar for other distance measures and *q* values (see Text and table 1 and supplementary tables S8–11, Supplementary Material online). Diversity of non-APGs (covariate) is presented as a color gradient. The PGLS regression line with *MHC-I* slope from the model, including *MHC-I* and non-APG as predictors is shown for the models with a significant *MHC-I* effect. (*A*) Relationship between *MHC-I* diversities and APGs diversities averaged over all APGs, (*B* and *C*) Relationship between individual-level (α, *B*) or species-wide (γ, *C*) diversity of *MHC-I* and three functional subsets of APGs, *PSMBs*: *PSMB8* and *PSMB9*, *TAPs*: *TAP1* and *TAP2*.

Diversities within all three categories of genes investigated here, that is, *MHC-I*, APGs, and non-APGs varied, sometimes by orders of magnitude, among salamander species (figs. 1 and 4; supplementary table S8, Supplementary Material online). *MHC-I* diversity was generally much higher than diversity of either APGs or non-APGs. The differences in the number of *MHC-I* alleles per individual apparently reflect interspecific differences in the extent of gene duplication and intraspecific copy number variation (supplementary table S3, Supplementary Material online). APG diversity in turn was generally higher than that of non-APGs, *PSMBs*, and *TAPs* exhibited comparable diversities, whereas *TAPBP* was less diverse (fig. 4 and supplementary table S8, Supplementary Material online).

We explored the relationships between *MHC-I* and APG diversity, simultaneously controlling for non-APG diversity, using a series of PGLS models. In the analysis not including APGs, diversity of non-APGs did not explain *MHC-I* diversity (table 2 and supplementary tables S9–S11, Supplementary Material online). The general formulation of the coevolution hypothesis, as applied here, considers all five APGs as a single class and was tested accordingly, using as the response variable the unweighted average of all APG diversities within species. The APGs may, however, also be considered a heterogeneous group consisting of three functional subgroups: *PSMBs* (*PSMB8* and *PSMB9*), *TAPs* (*TAP1* and *TAP2*), and *TAPBP*. As the strength of coevolution with *MHC-I* may differ among subgroups, or only some of APGs may coevolve with *MHC-I*, we also fitted models using each subgroup and individual APGs as the response variable. The results were broadly similar for within-individual (α) and species-wide (γ) diversity, though the signal was stronger for the latter (fig. 4 and table 2).

**Table 2.** Summary of PLGS Models for Diversities at the Amino Acid Level.

		Within-Individual (α) Diversity (*q *=* *0)			Species-Wide (γ) Diversity (*q *=* *1)		
Response Variable	Parameter	Estimate	SE	*P*-Val	Estimate	SE	*P*-Val
APGs	df* *=* *3, 27	*R* ^2^ * *=* *0.059 (*P *=* *0.17), λ * *=* *0.41			*R^2^ = 0.300 (P = 0.003), λ = 0.78*		
	Intercept	0.024	0.009	0.012	0.028	0.018	0.139
	*MHC-I*	0.006	0.005	0.226	*0.020*	*0.006*	*0.003*
	Non-APG	0.702	0.446	0.127	0.641	0.529	0.237
*PSMB8*	df* *=* *3, 19	*R* ^2^ * *=* *0 (*P *=* *0.45), λ* *=* *0.00			*R* ^2^ * *=* *0.098 (*P *=* *0.146), λ* *=* *0.49		
	Intercept	0.021	0.016	0.215	0.033	0.028	0.247
	*MHC-I*	0.008	0.009	0.395	0.024	0.013	0.071
	Non-APG	1.065	0.927	0.265	0.403	0.929	0.670
*PSMB9*	df* *=* *3, 25	*R* ^2^ * *=* *0 (*P *=* *0.84), λ* *=* *0.00			*R* ^2^ * *=* *0.048 (*P *=* *0.208), λ* *=* *0.00		
	Intercept	0.047	0.017	0.009	0.027	0.024	0.264
	*MHC-I*	−0.004	0.010	0.680	0.023	0.013	0.084
	Non-APG	−0.447	0.973	0.650	−0.128	1.095	0.908
*PSMBs*	df* *=* *3, 25	*R* ^2^ * *=* *0 (*P *=* *0.95), λ* *=* *0.00			*R* ^2^ * *=* *0.085 (*P *=* *0.125), λ* *=* *0.92		
	Intercept	0.044	0.013	0.003	0.052	0.033	0.129
	*MHC-I*	−0.001	0.008	0.879	0.019	0.009	0.054
	Non-APG	−0.236	0.793	0.769	−0.743	0.796	0.352
*TAP1*	df* *=* *3, 27	*R* ^2^ * *=* *0.136 (*P *=* *0.053), λ* *=* *1.00			*R^2^ = 0.567* (*P = 5 × 10^−6^*), *λ = 1.00*		
	Intercept	0.012	0.014	0.395	0.007	0.020	0.740
	*MHC-I*	*0.013*	*0.006*	*0.033*	*0.026*	*0.004*	*5 × 10^−6^*
	Non-APG	0.469	0.343	0.183	*1.072*	*0.365*	*0.007*
*TAP2*	df* *=* *3, 26	*R^2^ = 0.345* (*P = 0.002*), *λ = 0.68*			*R^2^ = 0.298* (*P = 0.004*), *λ = 0.67*		
	Intercept	0.018	0.012	0.153	0.033	0.025	0.199
	*MHC-I*	*0.015*	*0.006*	*0.012*	*0.021*	*0.009*	*0.030*
	Non-APG	*1.667*	*0.517*	*0.003*	*1.891*	*0.816*	*0.029*
*TAPs*	df* *=* *3, 27	*R^2^ = 0.392* (*P = 0.0005*), *λ = 0.79*			*R^2^ = 0.455* (*P = 0.0001*)*, λ = 0.75*		
	Intercept	0.015	0.010	0.122	0.020	0.019	0.283
	*MHC-I*	*0.012*	*0.004*	*0.008*	*0.024*	*0.007*	*0.001*
	Non-APG	*1.335*	*0.367*	*0.001*	*1.494*	*0.565*	*0.013*
*TAPBP*	df* *=* *3, 27	*R^2^ = 0.281* (*P = 0.004*), *λ = 0.30*			*R^2^ = 0.228* (*P = 0.012*), *λ = 0.00*		
	Intercept	0.000	0.004	0.740	0.001	0.009	0.909
	*MHC-I*	0.003	0.002	0.147	0.004	0.005	0.467
	Non-APG	*0.772*	*0.227*	*0.002*	*1.292*	*0.453*	*0.008*
*MHC-I*	df* *=* *2, 28	*R* ^2^ * *=* *0.00 (*P *=* *0.74), λ* *=* *1.00			*R* ^2^ * *=* *0.039 (*P *=* *0.15), λ* *=* *0.00		
	Intercept	1.256	0.427	0.007	1.273	0.251	0.00002
	Non-APG	−3.901	11.644	0.740	24.360	16.506	0.151

Note.—Phylogenetic least squares models tested whether diversity of APGs (response variable, considered as a group, as functional subsets, or as individual genes) was explained by *MHC-I* and non-APG diversity. For each model, the overall *R*^2^ and *P* value are given as well as the estimates of model parameter (Parameter), their standard errors (SE), and associated significance (*P*-val). At the bottom of the table the results of the model that tested whether *MHC-I* diversity was explained by non-APG diversity, so this model did not include APGs. The strength of phylogenetic signal was measured with Pagel’s λ (λ). Significant results are in italics. Other models are summarized in supplementary tables S9–S11, Supplementary Material online. APGs, mean diversity of all APGs as response variable; *PSMBs*, mean *PSMB8* and *PSMB9* diversity as response variable; *TAPs*, mean *TAP1* and *TAP2* diversity as response variable; df, degrees of freedom.

*MHC-I* α diversity was unrelated to mean APG α diversity for any distance measure, whereas a significant effect of non-APGs was only detected for synonymous divergence (*P *=* *0.013, table 2 and supplementary table S9, Supplementary Material online). When the three APG subsets were analyzed separately, *TAP* α diversity was explained by both *MHC-I* (*P *=* *0.008 for both amino acid *p*-distance and Grantham distance, the effect for synonymous divergence was marginally nonsignificant) and non-APG α diversities (*P *≤* *0.002 for all three distances). We did not detect an effect of *MHC-I* on *PSMB* or *TAPBP* α diversity and the effect of non-APGs was significant only for *TAPBP* diversity measured with the amino acid *p*-distance (*P *=* *0.002).

*MHC-I* γ diversity was positively related to mean APG γ diversity. This effect was significant for the amino acid *p*-distance and Grantham distance for all weightings (*q* values) of allele frequencies (all *P *≤* *0.006), and for synonymous diversity for *q *=* *0 and 1 (*P *=* *0.002 and 0.027, respectively, table 2 and supplementary table S9, Supplementary Material online). The relationship between non-APG and mean APG γ diversity was significant only for synonymous variation (all *P *≤* *0.001). Similarly to α diversity, there was also considerable heterogeneity between individual APGs and their functionally related subsets in γ diversity. Although the positive relationship between *MHC-I* and *TAP* γ diversity was strong (stronger for *TAP1*), for *PSMBs*, it was weak and patchy—reaching significance only for some combinations of *q* and distance measures, and no effect was detected for *TAPBP* (table 2 and supplementary table S9, Supplementary Material online). The relationship between non-APG γ diversity and γ diversity of particular APGs also varied, with the strongest effect for synonymous diversity in *TAPs* (supplementary table S9, Supplementary Material online). The results of PGLS modeling for sample sizes standardized to 15 individuals per species, and for the “conserved anchor” data set were very similar to the results obtained for the full data set (supplementary tables S10 and S11, Supplementary Material online).

## Discussion

Both *MHC-I* and APG diversity vary widely among salamander species, making them a suitable system for testing predictions of the coevolution hypothesis. *MHC-I* diversity predicted species-wide—but not within-individual—mean APG diversity in the PGLS analysis. The analysis of functional APG subcategories and individual APGs showed that the signal is driven by *TAP1* and *TAP2*, with a significant *MHC-I* effect at both the within-individual and species-wide levels. No consistent effect was detected for *PSMBs*, and, especially, *TAPBP*. Thus, *TAPs*, but not other APGs robustly support the major prediction of the coevolution hypothesis tested in the current study. This is in line with the results of Palomar et al. (2021) who examined signatures of adaptive evolution in salamander APGs at the phylogenetic scales, as predicted under coevolution, and found more signal of adaptive evolution in *TAPs* than in other APGs.

The pattern of a positive correlation between *MHC-I* and *TAP* diversity suggests that coevolution does not select against the expansion of the *MHC-I* family. The establishment of multiple highly expressed *MHC-I* genes following duplication may be allowed or even favored, as long as all MHC-I variants encoded on a haplotype efficiently bind peptides pumped by the TAP variant(s) encoded on it (Kaufman 1999; Palomar et al. 2021). The number of functional *MHC-I* genes on a haplotype would not be strongly constrained under this scenario, whereas their postduplication divergence could be. Such a situation has been observed in plant *rbcS* encoding the small subunit of RuBisCO enzyme—postduplication divergence of *rbcS* copies has been strongly constrained by the requirements of coevolution with the large subunit (Yamada et al. 2019). The number of *MHC-I* genes differs considerably among salamander species. This, together with copy number variation within species, indicates that the number of *MHC-I* genes can change rapidly in response to as yet incompletely understood selection pressures and complex tradeoffs (O’Connor et al. 2018; Phillips et al. 2018; Migalska et al. 2019; Radwan et al. 2020). Gene duplication also occurs in APGs, but is more limited (Palomar et al. 2021, this study). Thus, the range of *MHC-I* diversity within haplotypes (which correlates with within-individual diversity) would vary more among species than would the APG diversity, potentially leading to a weaker correlation at the within-individual compared with the species-wide level. Species-wide diversity of both *MHC-I* and APGs would be determined by the number and frequencies of different haplotypes carrying various coadapted *MHC-I–*APGs combinations segregating within the species, leading to the observed correlation between *MHC-I* and APG species-wide diversities.

The strongest support for *MHC-I–*APG coevolution in salamanders was found in *TAPs*, which is consistent with evidence from several species that have been studied in-depth: chicken (Walker et al. 2011), rat (Joly et al. 1998), *Xenopus* (Ohta et al. 2003), and zebrafish (McConnell et al. 2016). Our test of the coevolution hypothesis in a comparative framework implies that coevolution between at least *MHC-I* and *TAPs* may be widespread in other taxa with duplicated *MHC-I* genes. To confirm whether this is the case, additional comparative studies in other taxonomic groups, using methodology similar to that applied here, are needed. Candidate taxa for such additional analyses include teleost fishes (Grimholt et al. 2015), squamate reptiles (Radwan et al. 2014; Olivieri et al. 2020), and several orders of birds, particularly the Passeriformes (He et al. 2021). If coevolution is widespread, *MHC-I* diversity should explain *TAP* diversity in these taxa as well.

In contrast to *TAP* results, we found only a weak and inconsistent relationship between *MHC-I* and *PSMB* diversity. Indeed, the available evidence for coevolution of *PSMBs* is based mainly on a tight linkage with *TAPs* and cosegregation of divergent lineages of both genes in the frog *Xenopus* (reviewed in Kasahara and Flajnik [2019]), whereas no functional data supporting coevolution are available. In fact, birds lack immunoproteasome (Erath and Groettrup 2015), so no conclusive evidence could have been provided by the otherwise extensive functional research on coevolution in chicken (reviewed in Kaufman [2015]). One striking feature of nonmammalian *PSMBs*, which has been attributed to possible coevolution with different *MHC-I* alleles, still awaits explanation. Many species possess two *PSMB8* lineages that most likely differ in catalytic properties and have apparently been maintained over evolutionary timescales by extremely strong balancing selection (Huang et al. 2013). The two *PSMB8* lineages also occur in the Urodela, and we also detected two distinct *PSMB9* lineages in the family Salamandridae, although they were less divergent than those of *PSMB8* (Palomar et al. 2021). The relationship between *PSMB* lineages, the overall diversity of these genes, and *MHC-I* in salamanders remains unresolved. Although tight linkage creates conditions for coevolution, our data suggest some independence of the balancing selection mechanisms acting on *PSMBs* and *MHC-I*. Recent findings revealed diverse roles of the immunoproteasome, apart from providing antigenic peptides to the MHC-I (reviewed in Ferrington and Gregerson [2012] and Murata et al. [2018]). These include control of transcriptional activation and modulation of downstream cytokines, a role in T cell differentiation, involvement in the response to oxidative stress and a role in protein homeostasis during inflammation, a role in lipid metabolisms, and even some function in uninjured, immunoprivileged tissues such as the retina and brain. Potentially, any of these functions could contribute to the maintenance of divergent *PSMB* lineages.

*TAPBP* diversity did not correlate with that of *MHC-I*, whereas both within-individual and species-wide amino acid diversity of this gene was explained to some extent by the diversity of non-APGs. This suggests that *TAPBP* variation is determined by the joint action of purifying selection and demography, as elsewhere in the genome (Ellegren and Galtier 2016). *TAPBP* also consistently showed the lowest diversity among the five APGs. Thus, comparative data do not support coevolution between *MHC-I* and *TAPBP* in salamanders. Perhaps the recombination distance between *TAPBP* and *MHC-I* (∼0.45 cM vs. <0.1* *cM between the remaining four APGs and *MHC-I* in *Lissotriton*, the only salamander with the necessary data available, Palomar et al. 2021) is too large for coevolution to occur. In addition, MHC-I alleles differ greatly in their dependence on TAPBP for high-affinity peptide loading (Peh et al. 1998; van Hateren et al. 2013; Raghavan and Geng 2015). This can further obscure a signal of coevolution, because the presence of numerous MHC-I alleles that do not rely on TAPBP could decouple *MHC-I* and *TAPBP* diversities. So far the only evidence for coevolution, found in chicken, is based on interactions of polymorphic TAPBP with MHC-I in an allele-specific manner (van Hateren et al. 2013). However, this could be system-specific: chicken has only one highly expressed *MHC-I* gene within an extremely tightly linked MHC region. Unfortunately almost no other information on *TAPBP* polymorphism is available in nonmammalian systems.

The phylogenetic correlation between *MHC-I* and *TAP* diversity reported here is statistically robust, as it holds across allele frequency weightings (*q* values), measures of genetic distance, and regardless of whether sample sizes were standardized across species. However, we also need consider carefully explanations other than coevolution for the observed relationship between *MHC-I* and *TAP* diversity. One could argue that the correlation is a simple consequence of tight physical linkage between *MHC-I* and *TAPs*, so that the processes that promote *MHC-I* duplication and divergence spill over to the neighboring genomic regions. The data, however, speak against such an interpretation. First, *TAPs* and *PSMBs* are extremely closely linked in newts (Palomar et al. 2021), whereas a robust correlation was detected only for the former. Second, we have controlled for genomic region-specific effects by including non-APGs alongside *MHC-I* and APGs.

Several factors may contribute to the moderate strength of the detected correlation. One is related to the recently elucidated differences in the diversity of peptides translocated and bound by different alleles of TAP and MHC-I, respectively (Chappell et al. 2015; Tregaskes et al. 2016; Kaufman 2018a). Although it was long recognized that the polymorphism of these molecules qualitatively affects peptide repertoires, it is now clear that the size and diversity of these repertoires also vary. Some molecules are “fastidious” (specialist), others “promiscuous” (generalist), promoting translocation/binding of few similar—or many diverse—peptides, respectively. The measures of diversity applied in the present study, based on sequence information only, might not fully capture these functional differences. In particular, generalists, fulfilling functions of a more diverse set of molecules, may erode the observed correlation.

Another potential complication is the possibility that nonfunctional pseudogenes or nonclassical genes may constitute a sizeable fraction of the reported *MHC-I* diversity. Not all *MHC-I* sequences detected in genomic DNA are highly expressed and pseudogenes are scattered across allele phylogenies in newts (Fijarczyk et al. 2018). These two observations, together with the monophyly of *MHC-I* generally observed at the level of families, suggest a rapid turnover of *MHC-I* genes, which likely includes both episodes of pseudogenization and recurrent emergence of nonclassical genes. The evolutionary dynamics of *MHC-I* probably contributes to the difficulties in establishing the classical status of *MHC-I* genes in salamanders based on sequence data alone (Sammut et al. 1999). Nonetheless, three major considerations argue against the possibility that nonfunctional or nonclassical genes are of major concern in our analyses. First, several *MHC-I* genes are similarly highly transcribed in multiple tissues of salamander species studied so far (Sammut et al. 1999; Fijarczyk et al. 2018; Palomar et al. 2021) and ubiquitous expression is consistent with their classical status. Although high expression at the mRNA level does not automatically imply high protein level or high cell surface expression (Tregaskes et al. 2016), it is suggestive of classical function. Second, alleles with signatures of pseudogenization constitute only a small fraction of salamander *MHC-I* diversity, whereas overall high polymorphism and consistent signal of positive selection, both hallmarks of classical *MHC* genes, has been detected in all species studied to date (Fijarczyk et al. 2018; Minias et al., in preparation). Third, the PGLS analyses using the “conserved anchor” *MHC-I* data set produced results very similar to those including all potentially functional *MHC-I* alleles. To summarize, although we cannot completely rule out that certain nonfunctional or nonclassical *MHC-I* alleles were included in our analyses, they are unlikely to contribute to the signal of phylogenetic correlation between *MHC-I* and APG diversities. Instead, they may have introduced noise, which would not affect our conclusions, but might also explain why the PGLS models had only a moderate predictive power.

Our approach has some limitations, as phylogenetic comparative analysis, although suggestive, does not provide direct evidence of causality. It does, however clearly indicate the need for mechanistic tests in taxa with polymorphic APGs (in particular *TAPs*) and duplicated classical *MHC-I* genes. Such tests should examine the binding profiles of MHC-I proteins encoded on a single haplotype and compare them with TAP transport specificities. Under coevolution the binding profiles of MHC-I variants encoded on the same haplotype would be more similar than those of MHC-I variants encoded on different haplotypes and would match the haplotype’s TAP pumping specificity. To date, such experiments have been performed in chicken (Walker et al. 2011; Tregaskes et al. 2016), which has one highly expressed MHC-I molecule (encoded by *BF2* gene), one poorly expressed (encoded by *BF1*), and TAP pumping specificity matching the antigen-binding specificity of the highly expressed *BF2* gene product (supporting coevolution with just a single *MHC-I* gene). The next, challenging step would be to expand the scope of this approach to species with duplicated highly expressed and polymorphic *MHC-I*. Such an endeavor would require advanced experimental tools including homozygous strains with well-defined *MHC* haplotypes carrying multiple classical class I genes and corresponding cell lines. Such resources are increasingly available for various taxa that possess multiple *MHC-I* genes, including not only zebrafish and passerine birds, but also salamanders, such as *Pleurodeles waltl* and the axolotl (Reiß et al. 2015; Elewa et al. 2017). We hope that recent advancements in molecular biology, including those facilitating directed mutagenesis and generation of transgenic and knockout lines, will prompt mechanistic tests that will be capable of supporting or rejecting haplotype-specific coevolution of APGs with multiple *MHC-I*s.

## Conclusions

Here, we report the first comparative test of a crucial prediction of the coevolution hypothesis—a positive phylogenetic correlation between *MHC-I* and APG diversities. Data from 30 salamander species across six families support this prediction, with the support restricted mainly to one APG subclass—*TAPs*. Our results imply that coevolution does not prevent the expansion of *MHC-I* gene family, although it may restrict postduplication divergence of *MHC-I* genes. Nonmammalian vertebrates thus may be able to respond to diverse selection pressures by rapidly expanding or contracting the *MHC-I* gene family, while retaining the benefits of coevolution between *MHC-I* and *TAPs* within haplotypes. Such a mechanism would provide a great deal of flexibility in shaping the adaptive immune response.

## Materials and Methods

### Laboratory Procedures

DNA was extracted using the Wizard Genomic DNA Purification Kit (Promega). *MHC-I* exons 2 and 3 as well as *BRD2* variation was assessed using amplicon sequencing, whereas diversity of the remaining genes was examined by resequencing, following target capture with overlapping MIPs (Niedzicka et al. 2016). MIPs and primers for amplification of *MHC-I* and *BRD2* (supplementary table S12, Supplementary Material online) were designed from available salamander transcriptomes (Palomar et al. 2021). For the purpose of the current study, we additionally generated tailtip transcriptomes of *Eurycea bislineata*, *Desmognathus fuscus*, and *Proteus anguinus* and assembled transcriptome of *Plethodon cinereus* using RNAseq data deposited in SRA (SRR9925250, SRR9925255, SRR9925273, and SRR9925296). Details of primer and MIP design, laboratory procedures, Illumina sequencing, SNP-calling, and genotyping are in supplementary methods, Supplementary Material online.

### Identification of Putative Functional *MHC-I* Sequences

In nonmodel species that possess duplicated *MHC-I* genes, locus-specific primers for amplification of the variable exons usually cannot be designed and alleles from multiple loci are coamplified. Given sufficient similarity, gene fragments, pseudogenes, or other similar genes can also be amplified. To include in our analyses only potentially functional *MHC-I* alleles, we first removed all sequences with signatures of pseudogenization—frameshifts or internal stop codons. The remaining alleles could still be derived from both classical and nonclassical *MHC-I* genes. As almost all *MHC-I* alleles in *Lissotriton* newts segregate as stable haplotypes (Palomar et al. 2021), classical and nonclassical genes in salamanders are most likely tightly linked. Distinguishing between these two categories of MHC genes on the basis of sequence alone is challenging, probably even more so in salamanders; in the axolotl, sequences with intermediate characteristics between classical and nonclassical *MHC-I* have been described (Sammut et al. 1999). To minimize the risk that our results are distorted by the inclusion of nonclassical *MHC-I* sequences, in addition to the data set comprising all putative functional alleles, we also prepared a smaller “conserved anchor” data set, including only sequences that in key residues that anchor the termini of the antigenic peptide contained the amino acids conserved in most classical MHC-I molecules (Kaufman et al. 1994; Sammut et al. 1999). These were Y59(61) in exon 2, and T143(47), K146(50) or R146(50), W147(51), and Y159(66) in exon 3; the numbers following the letter indicate positions in the HLA-A molecule and the numbers in parentheses the positions in our alignments (supplementary fig. S1, Supplementary Material online). By applying this approach, we probably removed also many classical alleles, as in the phylogenetic trees (not shown) only some alleles excluded from the “conserved anchor” data set formed clusters that may represent nonclassical genes, whereas other alleles were scattered on the tree and intermixed with the “conserved anchor” alleles. Therefore, the “conserved anchor” data set is probably a conservative, “worst-case” scenario.

### Genetic Diversity

The overall diversity of salamander *MHC-I* alleles was visualized, separately for exons 2 and 3, with BIONJ (Gascuel 1997) trees constructed from the matrix of Jukes–Cantor distances among DNA sequences of all potentially functional alleles. Alleles were color-coded by family to visualize the extent to which alleles clustered together within salamander families and assess whether orthology is retained over extended evolutionary periods.

A proper test of the phylogenetic correlation between *MHC-I* and APG diversities requires appropriate diversity measures, and we were interested in both within-individual and species-wide diversity. Because most of the studied genes were duplicated in at least some species and the extent of duplication differed among genes and taxa, standard population genetic measures, such as nucleotide diversity, were not appropriate. Instead, we adopted measures of α and γ diversity developed in ecology to study species diversity, but increasingly used also for measuring genetic diversity (Sherwin et al. 2017; Gaggiotti et al. 2018). We used Hill numbers-based phylogenetic diversity as defined by Chao et al. (2014). This approach has three major advantages: 1) it naturally accommodates an arbitrary level of gene duplication; 2) it allows an assessment of the effect of rare and common alleles on diversity within a single framework by varying the *q* value: *q *=* *0 assigns equal weight to all variants, regardless of their frequency, and diversity corresponds to the number of alleles, *q *=* *1 weights variants according to their frequency and diversity corresponds to the exponential of Shannon’s diversity index, and *q *=* *2 gives more weight to frequent variants resulting in the inverse of Simpson’s diversity index; and 3) it allows the application of various measures of genetic distance among alleles/haplotypes, including those most relevant for MHC diversity, such as synonymous DNA divergence or functional protein divergence. In this approach haplotypes/alleles were analogous to species, individuals to local communities and all individuals sampled within species to the total community. Thus, phylogenetic α diversity was the sum of branch lengths connecting an individual’s alleles in the phylogeny of a given locus, providing a measure of within-individual diversity. Species-wide diversity was estimated as the phylogenetic γ diversity, the sum of branch lengths in the phylogeny of all alleles detected in a species, with various allele frequency weighting schemes applied by varying *q*, as described in Chao et al. (2014).

To calculate within-individual (α) diversity, we used information on allele presence–absence, not attempting to estimate the number of copies of each allele. Hence, α diversities were calculated and interpreted only for *q *=* *0. We deem this approach appropriate, as codominant expression of APGs and *MHC-I* generally results in their dominant effect on fitness, that is, it is the presence of the allele in an individual, not its number of copies that matters (Radwan et al. 2020). Because the genotype of each individual was known with an accuracy up to the genotyping error, so was its α diversity. The situation with species-wide (γ) diversity is slightly more complicated. First, different weightings of allele frequencies (*q *=* *0, 1, 2) provide complementary information so we report and interpret them all. Second, γ diversity depends on sample size, with the strongest dependence at *q *=* *0. Differences among species in sample sizes should not generally be a problem in testing evolutionary correlations, as long as they do not differ across categories of genes within species, which was generally the case in our study. Nonetheless, we also calculated diversities for sample sizes standardized to the minimum available for all species—15 individuals, as determined by the sample size of *Andrias davidianus*. To minimize missing data, the 15 individuals of each species with the highest coverage in MIPs were included.

Because both amplicon and MIP resequencing produced stacks of overlapping paired-end reads starting at defined positions, we performed the diversity analysis at the level of physically phased microhaplotypes (fig. 3*A*). For *MHC-I* exons and *BRD2*, haplotypes were reconstructed during genotyping with AmpliSAS (Sebastian et al. 2016). The remaining genes were divided into nonoverlapping segments and microhaplotypes were obtained for each segment separately using the R package *microhaplot* (Ng 2019) and custom R scripts, as described in supplementary methods, Supplementary Material online. Then, phylogenetic within-individual (α) and species-wide (γ) diversities were calculated for each segment. α diversity was the sum of the phylogenetic tree branch lengths connecting the segment’s haplotypes within the individual, whereas γ diversity was the length of the branches connecting all segment’s haplotypes within species, weighted accordingly for various *q* values (fig. 3*B*; Chao et al. 2014). To obtain the per-base/per-amino acid estimate for each gene, the weighted average of segment diversities was calculated, with segments weighted according to their lengths (fig. 3*C*).

We used the following measures of sequence divergence: 1) nucleotide divergence at synonymous codon positions estimated using the method of Li (1993), which should be affected mainly by demography, 2) protein divergence estimated with the amino acid *p*-distance, and 3) protein divergence estimated using the Grantham (1974) distance, which was shown to adequately reflect functional divergence between human MHC alleles (Pierini and Lenz 2018). BIONJ trees were constructed for each segment (see above) from the matrices of genetic distances using *ape* (Paradis and Schliep 2019) and diversities were calculated in *hillR* (Li 2018). All analyses were performed in R.

### Statistical Analysis

The coevolution hypothesis evaluated in this study predicts a positive correlation between APG and *MHC-I* diversity when controlling for non-APG (a “covariate”) diversity. We tested this prediction using PGLS in *caper* (Orme et al. 2013), in order to take into account the nonindependence of related species. The model including APG diversity as the response variable and *MHC-I* and non-APG diversity as continuous predictors was fitted with the *pgls* function. The nonindependence between residuals was modeled using the maximum likelihood estimate of Pagel’s λ to transform the variance–covariance matrix obtained from the phylogeny under the Brownian motion model (Freckleton et al. 2002). Separate models were fitted for α and γ diversity. Because there was no a priori reason to transform the data and a visual inspection of residuals did not reveal serious departures from normality, we used untransformed diversity estimates in PGLS modeling.

We used the time calibrated phylogeny of Jetz and Pyron (2018), to which we added the recently recognized species *O. nesterovi* (van Riemsdijk et al. 2017). We also used a modified phylogeny with the topology and divergence times within Salamandridae taken from newer phylogenomics-based phylogenies of the family Salamandridae (Rancilhac et al. 2021) and the genus *Triturus* (Wielstra et al. 2019). The PGLS modeling results with both phylogenies were virtually identical, so we present only the former.

## Supplementary Material

Supplementary data are available at *Molecular Biology and Evolution* online.

## Supplementary Material

msab237_Supplementary_Data

## References

[msab237-B1] Adams EJ , Luoma AM. 2013. The adaptable major histocompatibility complex (MHC) fold: structure and function of nonclassical and MHC class I–like molecules. Annu Rev Immunol. 31:529–561. 23298204 10.1146/annurev-immunol-032712-095912

[msab237-B2] Banach M , Edholm E-S, Robert J. 2017. Exploring the functions of nonclassical MHC class Ib genes in *Xenopus laevis* by the CRISPR/Cas9 system. Dev Biol. 426(2):261–269. 27318386 10.1016/j.ydbio.2016.05.023 PMC5501940

[msab237-B3] Blees A , Januliene D, Hofmann T, Koller N, Schmidt C, Trowitzsch S, Moeller A, Tampé R. 2017. Structure of the human MHC-I peptide-loading complex. Nature 551(7681):525–528. 29107940 10.1038/nature24627

[msab237-B4] Blum JS , Wearsch PA, Cresswell P. 2013. Pathways of antigen processing. Annu Rev Immunol. 31:443–473. 23298205 10.1146/annurev-immunol-032712-095910 PMC4026165

[msab237-B5] Braud VM , Allan DS, Wilson D, McMichael AJ. 1998. TAP-and tapasin-dependent HLA-E surface expression correlates with the binding of an MHC class I leader peptide. Curr Biol. 8(1):1–10. 9427624 10.1016/s0960-9822(98)70014-4

[msab237-B6] Chao A , Chiu C-H, Jost L. 2014. Unifying species diversity, phylogenetic diversity, functional diversity, and related similarity and differentiation measures through Hill numbers. Annu Rev Ecol Evol Syst. 45(1):297–324.

[msab237-B7] Chappell PE , Meziane EK, Harrison M, Magiera Ł, Hermann C, Mears L, Wrobel AG, Durant C, Nielsen LL, Buus S, et al 2015. Expression levels of MHC class I molecules are inversely correlated with promiscuity of peptide binding. Elife 4:e05345. 25860507 10.7554/eLife.05345 PMC4420994

[msab237-B8] Drews A , Westerdahl H. 2019. Not all birds have a single dominantly expressed MHC-I gene: transcription suggests that siskins have many highly expressed MHC-I genes. Sci Rep. 9(1):19506–19511. 31862923 10.1038/s41598-019-55800-9 PMC6925233

[msab237-B9] Edholm E-S , Goyos A, Taran J, Andino FDJ, Ohta Y, Robert J. 2014. Unusual evolutionary conservation and further species-specific adaptations of a large family of nonclassical MHC class Ib genes across different degrees of genome ploidy in the amphibian subfamily Xenopodinae. Immunogenetics 66(6):411–426. 24771209 10.1007/s00251-014-0774-5 PMC4096976

[msab237-B10] Edholm E-S , Saez L-MA, Gill AL, Gill SR, Grayfer L, Haynes N, Myers JR, Robert J. 2013. Nonclassical MHC class I-dependent invariant T cells are evolutionarily conserved and prominent from early development in amphibians. Proc Natl Acad Sci U S A. 110(35):14342–14347. 23940320 10.1073/pnas.1309840110 PMC3761575

[msab237-B11] Elewa A , Wang H, Talavera-López C, Joven A, Brito G, Kumar A, Hameed LS, Penrad-Mobayed M, Yao Z, Zamani N. 2017. Reading and editing the *Pleurodeles waltl* genome reveals novel features of tetrapod regeneration. Nat Commun. 8:1–9. 29273779 10.1038/s41467-017-01964-9 PMC5741667

[msab237-B12] Ellegren H , Galtier N. 2016. Determinants of genetic diversity. Nat Rev Genet. 17(7):422–433. 27265362 10.1038/nrg.2016.58

[msab237-B13] Erath S , Groettrup M. 2015. No evidence for immunoproteasomes in chicken lymphoid organs and activated lymphocytes. Immunogenetics 67(1):51–60. 25403261 10.1007/s00251-014-0814-1

[msab237-B14] Ferrington DA , Gregerson DS. 2012. Immunoproteasomes: structure, function, and antigen presentation. Prog Mol Biol Transl Sci. 109:75–112. 22727420 10.1016/B978-0-12-397863-9.00003-1 PMC4405001

[msab237-B15] Fijarczyk A , Dudek K, Niedzicka M, Babik W. 2018. Balancing selection and introgression of newt immune-response genes. Proc Roy Soc B. 285:20180819. 10.1098/rspb.2018.0819 PMC6111169 30111606

[msab237-B16] Fisette O , Schröder GF, Schäfer LV. 2020. Atomistic structure and dynamics of the human MHC-I peptide-loading complex. Proc Natl Acad Sci U S A. 117(34):20597–20606. 32788370 10.1073/pnas.2004445117 PMC7456110

[msab237-B17] Flajnik MF. 2018. A cold-blooded view of adaptive immunity. Nat Rev Immunol. 18(7):438–453. 29556016 10.1038/s41577-018-0003-9 PMC6084782

[msab237-B18] Freckleton RP , Harvey PH, Pagel M. 2002. Phylogenetic analysis and comparative data: a test and review of evidence. Am Nat. 160(6):712–726. 18707460 10.1086/343873

[msab237-B19] Frost DR. 2021. Amphibian species of the world: an online reference. Version 6.1. New York: American Museum of Natural History. Available from: https://amphibiansoftheworld.amnh.org/index.php.

[msab237-B20] Gaggiotti OE , Chao A, Peres‐Neto P, Chiu CH, Edwards C, Fortin MJ, Jost L, Richards CM, Selkoe KA. 2018. Diversity from genes to ecosystems: a unifying framework to study variation across biological metrics and scales. Evol Appl. 11(7):1176–1193. 30026805 10.1111/eva.12593 PMC6050189

[msab237-B21] Gascuel O. 1997. BIONJ: an improved version of the NJ algorithm based on a simple model of sequence data. Mol Biol Evol. 14(7):685–695. 9254330 10.1093/oxfordjournals.molbev.a025808

[msab237-B22] Grantham R. 1974. Amino acid difference formula to help explain protein evolution. Science 185(4154):862–864. 4843792 10.1126/science.185.4154.862

[msab237-B23] Grimholt U , Tsukamoto K, Azuma T, Leong J, Koop BF, Dijkstra JM. 2015. A comprehensive analysis of teleost MHC class I sequences. BMC Evol Biol. 15:1–17. 25888517 10.1186/s12862-015-0309-1 PMC4364491

[msab237-B24] He K , Minias P, Dunn PO. 2021. Long-read genome assemblies reveal extraordinary variation in the number and structure of MHC loci in birds. Genome Biol Evol. 13(2):evaa270. 33367721 10.1093/gbe/evaa270 PMC7875000

[msab237-B25] Horton R , Wilming L, Rand V, Lovering RC, Bruford EA, Khodiyar VK, Lush MJ, Povey S, Talbot CC, Wright MW, et al 2004. Gene map of the extended human MHC. Nat Rev Genet. 5(12):889–899. 15573121 10.1038/nrg1489

[msab237-B26] Huang C-H , Tanaka Y, Fujito NT, Nonaka M. 2013. Dimorphisms of the proteasome subunit beta type 8 gene (*PSMB8*) of ectothermic tetrapods originated in multiple independent evolutionary events. Immunogenetics 65(11):811–821. 23982299 10.1007/s00251-013-0729-2

[msab237-B27] Irisarri I , Baurain D, Brinkmann H, Delsuc F, Sire J-Y, Kupfer A, Petersen J, Jarek M, Meyer A, Vences M, et al 2017. Phylotranscriptomic consolidation of the jawed vertebrate timetree. Nat Ecol Evol. 1(9):1370–1378. 28890940 10.1038/s41559-017-0240-5 PMC5584656

[msab237-B28] Jetz W , Pyron RA. 2018. The interplay of past diversification and evolutionary isolation with present imperilment across the amphibian tree of life. Nat Ecol Evol. 2(5):850–858. 29581588 10.1038/s41559-018-0515-5

[msab237-B29] Joly E , Le Rolle AF, González AL, Mehling B, Stevens J, Coadwell WJ, Hünig T, Howard JC, Butcher GW. 1998. Co-evolution of rat TAP transporters and MHC class I RT1-A molecules. Curr Biol. 8(3):169–180. 9443915 10.1016/s0960-9822(98)70065-x

[msab237-B30] Kasahara M , Flajnik MF. 2019. Origin and evolution of the specialized forms of proteasomes involved in antigen presentation. Immunogenetics 71(3):251–261. 30675634 10.1007/s00251-019-01105-0 PMC6377343

[msab237-B31] Kaufman J. 1999. Co-evolving genes in MHC haplotypes: the “rule” for nonmammalian vertebrates? Immunogenetics 50(3–4):228–236. 10602883 10.1007/s002510050597

[msab237-B32] Kaufman J. 2015. Co‐evolution with chicken class I genes. Immunol Rev. 267(1):56–71. 26284471 10.1111/imr.12321

[msab237-B33] Kaufman J. 2018a. Generalists and specialists: a new view of how MHC class I molecules fight infectious pathogens. Trends Immunol. 39(5):367–379. 29396014 10.1016/j.it.2018.01.001 PMC5929564

[msab237-B34] Kaufman J. 2018b. Unfinished business: evolution of the MHC and the adaptive immune system of jawed vertebrates. Annu Rev Immunol. 36:383–409. 29677478 10.1146/annurev-immunol-051116-052450

[msab237-B35] Kaufman J , Milne S, Göbel TW, Walker BA, Jacob JP, Auffray C, Zoorob R, Beck S. 1999. The chicken B locus is a minimal essential major histocompatibility complex. Nature 401(6756):923–925. 10553909 10.1038/44856

[msab237-B36] Kaufman J , Salomonsen J, Flajnik M. 1994. Evolutionary conservation of MHC class I and class II molecules – different yet the same. Semin Immunol. 6(6):411–424. 7654997 10.1006/smim.1994.1050

[msab237-B37] Li D. 2018. hillR: taxonomic, functional, and phylogenetic diversity and similarity through Hill Numbers. J Open Source Softw. 3(31):1041.

[msab237-B38] Li W-H. 1993. Unbiased estimation of the rates of synonymous and nonsynonymous substitution. J Mol Evol. 36(1):96–99. 8433381 10.1007/BF02407308

[msab237-B39] Marjanović D , Laurin M. 2014. An updated paleontological timetree of lissamphibians, with comments on the anatomy of Jurassic crown-group salamanders (Urodela). Hist Biol. 26(4):535–550.

[msab237-B40] McConnell SC , Hernandez KM, Wcisel DJ, Kettleborough RN, Stemple DL, Yoder JA, Andrade J, de Jong JL. 2016. Alternative haplotypes of antigen processing genes in zebrafish diverged early in vertebrate evolution. Proc Natl Acad Sci U S A. 113(34):E5014–E5023. 27493218 10.1073/pnas.1607602113 PMC5003237

[msab237-B41] McConnell SC , Restaino AC, de Jong JL. 2014. Multiple divergent haplotypes express completely distinct sets of class I MHC genes in zebrafish. Immunogenetics 66(3):199–213. 24291825 10.1007/s00251-013-0749-y PMC3965299

[msab237-B42] Migalska M , Sebastian A, Radwan J. 2019. Major histocompatibility complex class I diversity limits the repertoire of T cell receptors. Proc Natl Acad Sci U S A. 116(11):5021–5026. 30796191 10.1073/pnas.1807864116 PMC6421458

[msab237-B43] Miura F , Tsukamoto K, Mehta RB, Naruse K, Magtoon W, Nonaka M. 2010. Transspecies dimorphic allelic lineages of the proteasome subunit β-type 8 gene (*PSMB8*) in the teleost genus *Oryzias*. Proc Natl Acad Sci U S A. 107(50):21599–21604. 21098669 10.1073/pnas.1012881107 PMC3003058

[msab237-B44] Müller V , De Boer RJ, Bonhoeffer S, Szathmáry E. 2018. An evolutionary perspective on the systems of adaptive immunity. Biol Rev Camb Philos Soc. 93(1):505–528. 28745003 10.1111/brv.12355

[msab237-B45] Murata S , Takahama Y, Kasahara M, Tanaka K. 2018. The immunoproteasome and thymoproteasome: functions, evolution and human disease. Nat Immunol. 19(9):923–931. 30104634 10.1038/s41590-018-0186-z

[msab237-B46] Ng T. 2019. microhaplot R package. Available from: https://github.com/ngthomas/microhaplot.

[msab237-B47] Niedzicka M , Fijarczyk A, Dudek K, Stuglik M, Babik W. 2016. Molecular inversion probes for targeted resequencing in non-model organisms. Sci Rep. 6:24051. 27046329 10.1038/srep24051 PMC4820773

[msab237-B48] Nonaka MI , Nonaka M. 2010. Evolutionary analysis of two classical MHC class I loci of the medaka fish, *Oryzias latipes*: haplotype-specific genomic diversity, locus-specific polymorphisms, and interlocus homogenization. Immunogenetics 62(5):319–332. 20174921 10.1007/s00251-010-0426-3

[msab237-B49] O’Connor EA , Cornwallis CK, Hasselquist D, Nilsson J-Å, Westerdahl H. 2018. The evolution of immunity in relation to colonization and migration. Nat Ecol Evol. 2(5):841–849. 29632357 10.1038/s41559-018-0509-3

[msab237-B50] Ohta Y , Flajnik MF. 2015. Coevolution of MHC genes (LMP/TAP/class Ia, NKT‐class Ib, NKp30‐B7H6): lessons from cold‐blooded vertebrates. Immunol Rev. 267(1):6–15. 26284468 10.1111/imr.12324 PMC4594805

[msab237-B51] Ohta Y , Goetz W, Hossain MZ, Nonaka M, Flajnik MF. 2006. Ancestral organization of the MHC revealed in the amphibian *Xenopus*. J Immunol. 176(6):3674–3685. 16517736 10.4049/jimmunol.176.6.3674

[msab237-B52] Ohta Y , Powis SJ, Lohr RL, Nonaka M, Pasquier LD, Flajnik MF. 2003. Two highly divergent ancient allelic lineages of the transporter associated with antigen processing (TAP) gene in *Xenopus*: further evidence for co‐evolution among MHC class I region genes. Eur J Immunol. 33(11):3017–3027. 14579270 10.1002/eji.200324207

[msab237-B53] Olivieri DN , Mirete-Bachiller S, Gambon-Deza F. 2020. MHC class I and II genes in Serpentes. *bioRxiv* 2020.06.12.133363.

[msab237-B54] Orme D , Freckleton R, Thomas G, Petzoldt T, Fritz S, Isaac N, Pearse W. 2013. The caper package: comparative analysis of phylogenetics and evolution in R. R Package Version. 5:1–36.

[msab237-B55] Palomar G , Dudek K, Wielstra B, Jockusch EL, Vinkler M, Arntzen JW, Ficetola GF, Matsunami M, Waldman B, Těšický M. 2021. Molecular evolution of antigen-processing genes in salamanders: do they coevolve with MHC class I genes? Genome Biol Evol. 13:evaa259. 33501944 10.1093/gbe/evaa259 PMC7883663

[msab237-B56] Paradis E , Schliep K. 2019. ape 5.0: an environment for modern phylogenetics and evolutionary analyses in R. Bioinformatics 35(3):526–528. 30016406 10.1093/bioinformatics/bty633

[msab237-B57] Peh CA , Burrows SR, Barnden M, Khanna R, Cresswell P, Moss DJ, McCluskey J. 1998. HLA-B27–restricted antigen presentation in the absence of tapasin reveals polymorphism in mechanisms of HLA class i peptide loading. Immunity 8(5):531–542. 9620674 10.1016/s1074-7613(00)80558-0

[msab237-B58] Phillips KP , Cable J, Mohammed RS, Herdegen-Radwan M, Raubic J, Przesmycka KJ, van Oosterhout C, Radwan J. 2018. Immunogenetic novelty confers a selective advantage in host–pathogen coevolution. Proc Natl Acad Sci U S A. 115(7):1552–1557. 29339521 10.1073/pnas.1708597115 PMC5816137

[msab237-B59] Pierini F , Lenz TL. 2018. Divergent allele advantage at human MHC genes: signatures of past and ongoing selection. Mol Biol Evol. 35(9):2145–2158. 29893875 10.1093/molbev/msy116 PMC6106954

[msab237-B60] Radwan J , Babik W, Kaufman J, Lenz TL, Winternitz J. 2020. Advances in the evolutionary understanding of MHC polymorphism. Trends Genet. 36(4):298–311. 32044115 10.1016/j.tig.2020.01.008

[msab237-B61] Radwan J , Kuduk K, Levy E, LeBas N, Babik W. 2014. Parasite load and MHC diversity in undisturbed and agriculturally modified habitats of the ornate dragon lizard. Mol Ecol. 23(24):5966–5978. 25355141 10.1111/mec.12984

[msab237-B62] Raghavan M , Geng J. 2015. HLA-B polymorphisms and intracellular assembly modes. Mol Immunol. 68(2 Pt A):89–93. 26239417 10.1016/j.molimm.2015.07.007 PMC4681577

[msab237-B63] Rancilhac L , Irisarri I, Angelini C, Arntzen JW, Babik W, Bossuyt F, Künzel S, Lüddecke T, Pasmans F, Sanchez E, et al 2021. Phylotranscriptomic evidence for pervasive ancient hybridization among Old World salamanders. Mol Phylogenet Evol. 155:106967. 33031928 10.1016/j.ympev.2020.106967

[msab237-B64] Reiß C , Olsson L, Hoßfeld U. 2015. The history of the oldest self‐sustaining laboratory animal: 150 years of axolotl research. J Exp Zool B Mol Dev Evol. 324(5):393–404. 25920413 10.1002/jez.b.22617

[msab237-B65] Robert J , Edholm E-S. 2014. A prominent role for invariant T cells in the amphibian *Xenopus laevis* tadpoles. Immunogenetics 66(9–10):513–523. 24898512 10.1007/s00251-014-0781-6

[msab237-B66] Sammut B , Du Pasquier L, Ducoroy P, Laurens V, Marcuz A, Tournefier A. 1999. Axolotl MHC architecture and polymorphism. Eur J Immunol. 29(9):2897–2907. 10508264 10.1002/(SICI)1521-4141(199909)29:09<2897::AID-IMMU2897>3.0.CO;2-2

[msab237-B67] Schloissnig S , Kawaguchi A, Nowoshilow S, Falcon F, Otsuki L, Tardivo P, Timoshevskaya N, Keinath MC, Smith JJ, Voss SR, et al 2021. The giant axolotl genome uncovers the evolution, scaling, and transcriptional control of complex gene loci. Proc Natl Acad Sci U S A. 118(15):e2017176118. 33827918 10.1073/pnas.2017176118 PMC8053990

[msab237-B68] Sebastian A , Herdegen M, Migalska M, Radwan J. 2016. amplisas: a web server for multilocus genotyping using next‐generation amplicon sequencing data. Mol Ecol Resour. 16(2):498–510. 26257385 10.1111/1755-0998.12453

[msab237-B69] Sherwin WB , Chao A, Jost L, Smouse PE. 2017. Information theory broadens the spectrum of molecular ecology and evolution. Trends Ecol Evol. 32(12):948–963. 29126564 10.1016/j.tree.2017.09.012

[msab237-B70] Tilloy F , Treiner E, Park S-H, Garcia C, Lemonnier F, De La Salle H, Bendelac A, Bonneville M, Lantz O. 1999. An invariant T cell receptor α chain defines a novel TAP-independent major histocompatibility complex class Ib–restricted α/β T cell subpopulation in mammals. J Exp Med. 189(12):1907–1921. 10377186 10.1084/jem.189.12.1907 PMC2192962

[msab237-B71] Tregaskes CA , Harrison M, Sowa AK, van Hateren A, Hunt LG, Vainio O, Kaufman J. 2016. Surface expression, peptide repertoire, and thermostability of chicken class I molecules correlate with peptide transporter specificity. Proc Natl Acad Sci U S A. 113(3):692–697. 26699458 10.1073/pnas.1511859113 PMC4725490

[msab237-B72] Trowitzsch S , Tampé R. 2020. Multifunctional chaperone and quality control complexes in adaptive immunity. Annu Rev Biophys. 49:135–161. 32004089 10.1146/annurev-biophys-121219-081643

[msab237-B73] van Hateren A , Carter R, Bailey A, Kontouli N, Williams AP, Kaufman J, Elliott T. 2013. A mechanistic basis for the co-evolution of chicken tapasin and major histocompatibility complex class I (MHC I) proteins. J Biol Chem. 288(45):32797–32808. 24078633 10.1074/jbc.M113.474031 PMC3820913

[msab237-B74] van Riemsdijk I , Arntzen JW, Bogaerts S, Franzen M, Litvinchuk SN, Olgun K, Wielstra B. 2017. The Near East as a cradle of biodiversity: a phylogeography of banded newts (genus *Ommatotriton*) reveals extensive inter-and intraspecific genetic differentiation. Mol Phylogenet Evol. 114:73–81. 28602572 10.1016/j.ympev.2017.05.028

[msab237-B75] Walker BA , Hunt LG, Sowa AK, Skjødt K, Göbel TW, Lehner PJ, Kaufman J. 2011. The dominantly expressed class I molecule of the chicken MHC is explained by coevolution with the polymorphic peptide transporter (TAP) genes. Proc Natl Acad Sci U S A. 108(20):8396–8401. 21536896 10.1073/pnas.1019496108 PMC3100931

[msab237-B76] Wielstra B , McCartney-Melstad E, Arntzen J, Butlin RK, Shaffer HB. 2019. Phylogenomics of the adaptive radiation of *Triturus* newts supports gradual ecological niche expansion towards an incrementally aquatic lifestyle. Mol Phylogenet Evol. 133:120–127. 30630099 10.1016/j.ympev.2018.12.032

[msab237-B77] Yamada K , Davydov II, Besnard G, Salamin N. 2019. Duplication history and molecular evolution of the rbcS multigene family in angiosperms. J Exp Bot. 70(21):6127–6139. 31498865 10.1093/jxb/erz363 PMC6859733

