# Supplementary material for: Coevolution between MHC Class I and Antigen-Processing Genes in Salamanders
Source: Mol Biol Evol. 2021 Aug 10;38(11):5092–106. doi: 10.1093/molbev/msab237 (PMC8557411; doi:10.1093/molbev/msab237)
Supplement: msab237_Supplementary_Data [file msab237_supplementary_data.zip › Palomar_et_al_Coevolution_Rev1_SM.pdf]

Supplementary Materials to

Coevolution between MHC class I and Antigen Processing Genes in salamanders

G. Palomar[1], K. Dudek[1], M. Migalska[1], J.W. Arntzen[2,3], G.F. Ficetola[4,5], D. Jelić[6], E. Jockusch[7], I. Martínez-Solano[8], M. Matsunami[9], H.B. Shaffer[10,11], J. Vörös[12], B. Waldman[13,14], B. Wielstra[2,3], *W. Babik[1]

[1]Institute of Environmental Sciences, Faculty of Biology, Jagiellonian University, Kraków, Poland; [2]Naturalis Biodiversity Center, P.O. Box 9517, 2300 RA leiden, Leiden, The Netherlands; [3]Institute of Biology Leiden, Leiden University, 2300 RA Leiden, The Netherlands; [4]Department of Environmental Sciences and Policy, University of Milano, Italy; [5]Laboratoire d'Ecologie Alpine (LECA), CNRS, Université Grenoble Alpes and Université Savoie Mont Blanc, Grenoble, France; [6]Croatian Institute for Biodiversity, Zagreb, Croatia; [7]Ecology and Evolutionary Biology, University of Connecticut, Storrs, CT USA; [8]Museo Nacional de Ciencias Naturales (MNCN), Consejo Superior de Investigaciones Científicas (CSIC), Madrid, Spain; [9]Department of Advanced Genomic and Laboratory Medicine, Graduate School of Medicine, University of the Ryukyus, Nishihara-cho, Japan; [10]Department of Ecology and Evolutionary Biology, University of California, Los Angeles, CA 90095, USA; [11]La Kretz Center for California Conservation Science, Institute of the Environment and Sustainability, University of California, Los Angeles, CA 90095, USA; [12]Department of Zoology, Hungarian Natural History Museum, Budapest, Hungary; [13]Department of Integrative Biology, Oklahoma State University, Stillwater, Oklahoma 74078, USA; [14]School of Biological Sciences, Seoul National University, Seoul 08826, South Korea

*correspondence: wieslaw.babik@uj.edu.pl
Institute of Environmental Sciences, Faculty of Biology, Jagiellonian University, Gronostajowa 7, 30-387 Kraków, Poland

Supplementary Methods

Primer and MIP design

Contigs representing *MHC-I*, APGs and nonAPGs were recovered from transcriptome assemblies using blast as described in Palomar et al. (2021). To design *MHC-I* primers we first identified conserved portions of exons 2 (Fijarczyk et al. 2018) and 3 suitable for primer location in the alignments of contigs. Then, we mapped the RNAseq from all available individuals to the *MHC-I* contigs and designed primers taking into account polymorphism information. To the extent possible, primers were designed to amplify the same fragments of *MHC-I* exons 2 and 3 across all genera (Table S12). Primers for amplification of a ca. 300 bp (excluding primers) fragment of a single *BRD2* exon were designed based on the alignment of *BRD2* contigs from all species.

All APGs and nonAPGs except *BRD2* were resequenced using Molecular Inversion Probes (MIPs, Niedzicka et al. 2016). To design MIPs we first identified exon boundaries using the axolotl and *Pleurodeles waltl* genome assemblies (Elewa et al. 2017; Nowoshilow et al. 2018). Then, we mapped RNAseq reads to APGs and nonAPGs contigs and identified SNPs using GATK UnifiedGenotyper (McKenna et al. 2010). We then used the information about exon boundaries and SNPs to design MIPs with MIPgen (Boyle et al. 2014), making sure that each MIP was contained within a single exon. A maximum of two polymorphisms per MIP arms was allowed; if more variation was present, we designed another MIP for the same target. Some MIPs designed for different species had identical sequences; in such cases the same probe was used for both species. Each species was analysed with its own MIPs pool, except a few species for which transcriptomes were not available: *Ommatotriton nesterovi* (MIPs from *O. ophryticus*), *Hynobius tokyoensis* (MIPs from *H. leechii*), and *Lissotriton italicus* (pool combining MIPs from *L. helveticus*, *L. montandoni*/*L. vulgaris* (Palomar et al. 2021) and *L. boscai*).

Targeted resequencing with Molecular Inversion Probes (MIPs)

Target capture and library construction were performed using the protocol described in Hiatt et al. (2013) with modifications. Probes were pooled equimolarly and 5'-phosphorylation was performed using 85 ul of the pool, 50 units of T4 Polynucleotide Kinase (NEB) and 10 ul of 10 × T4 DNA ligase buffer in a total volume of 100 ul. Reactions were incubated for 45 min at 37 °C, followed by inactivation of the kinase at 80 °C for 20 min. Captures were performed using 300–500 ng of genomic DNA, the phosphorylated probe pool at an approximately 1000-fold probe-to-target molar excess, and 1 ul of 10 × Ampligase DNA ligase buffer (Epicentre) in a total volume of 10 ul. The hybridization mixture was incubated at 98 °C for 3 min, 85 °C for 30

min, 60 °C for 60 min, and 56 °C for 120 min. Gap filling and ligation reactions contained 10 ul of hybridization mixture, 300 pm of each dNTPs (NEB), 20 nm NAD+ (NEB), 7.5 um betaine (Sigma), 1 ul of 10 × Ampligase DNA ligase buffer, 5 units of Ampligase DNA ligase (Epicentre) and 0.8 units of Phusion DNA polymerase (NEB) in a total volume of 20 ul. Reactions were incubated at 56 °C for 60 min and 72 °C for 20 min. Reactions were then cooled to 37 °C and 20 units of Exonuclease I (NEB) and 100 units of Exonuclease III (NEB) were added to degrade not circularized probes and genomic DNA. Reactions were incubated at 37 °C for 45 min and at 80 °C for 20 min. For each sample, PCR amplification of captured targets was performed using 1 unit of Phusion DNA polymerase (NEB), 0.5 uM of each indexed primer, Phusion buffer to 1x, 200 um each dNTPs (NEB), 10 ul of capture reaction and nuclease-free water to 50 ul. The following PCR conditions were used: 98 °C/30s, 30x (98 °C/10 s, 65 °C/30 s, 72 °C/30 s), 72 °C/10 min. PCR products from multiple samples were pooled equimolarly, run on a 1.5% agarose gel at 6.5 V/cm for 60 min and the band at ca. 270 bp was excised and purified using a Zymoclean Gel DNA Recovery Kit (Zymo). The purified PCR product was quantified via Qubit. The library was sequenced using custom primers on the MiSeq platform, producing 2 × 78 bp paired-end reads.

PCR amplification and sequencing

MHC-I and *BRD2* were PCR-amplified, PCR-free Illumina libraries were constructed and sequenced as described in Fijarczyk et al. (2018). Genotyping repeatability of exon 2 in plethodontid genera *Batrachoseps*, and *Eurycea* was not satisfactory, most likely due to stochasticity of amplification of some alleles. In these genera exon 2 was amplified in triplicates using two-step PCR. In PCR I specific primers (Table S12) had adaptors at their 5’ ends: F: 5’ACACTCTTTCCCTACACGACGCTCTTCCGATCT3’ and R: 5’GTGACTGGAGTTCAGACGTGTGCTCTTCCGATCT3’. Fifteen ul PCR reactions contained 7.5 ul of Multiplex PCR Master Mix (Qiagen), each primer at 1 uM and 50-100 ng of genomic DNA. The following PCR conditions were used: 95 °C/15 min, 35x (94 °C/30 s, 56 °C/30 s, 72 °C/60 s), 72 °C/10 min. The PCR product was purified using 0.8x AMPure XP beads and eluted with 7 ul of 0.1 x TE buffer. In PCR II universal indexed primers were used, i5: AATGATACGGCGACCACCGAGATCTACACNNNNNNNNNNACACTCTTTCCCTAC and i7: CAAGCAGAAGACGGCATACGAGATNNNNNNNNNNGTGACTGGAGTTCAG, where NNNNNNNNNN were the index sequences used to distinguish samples. Ten ul PCR reactions contained 7.5 ul of Multiplex PCR Master Mix (Qiagen), each primer at 1 uM and 2 ul of purified PCR I product. The following PCR conditions were used: 95 °C/15 min, 7x (94 °C/30 s, 56

°C/30 s, 72 °C/60 s), 72 °C/10 min. Two ul aliquots were run on 2% agarose gels to estimate band intensity, then amplicons were pooled equimolarly, pools were run on 2% agarose gels, the appropriate band was excised and purified using Zymoclean Gel DNA Recovery Kit (Zymo). The purified library was sequenced on Illumina MiSeq (2 x 220 bp reads).

Bioinformatic analysis of sequencing results

MIPs. We mapped raw reads using BWA-MEM algorithm with bwa-mips (Pedersen 2014), a wrapper around BWA (Li and Durbin 2009), which used coordinates from MIPgen to trim the arms with clipping penalty 100, 100 and mismatch penalty 2. Because not all arms were properly trimmed by bwa-mips we also applied additional trimming steps. Once all the reads had their arms trimmed, we extracted trimmed reads from bam files and merged reads in pairs with PEAR (Zhang et al. 2014). The resulting single-end fastq files were mapped to the reference using Bowtie2 (Langmead and Salzberg 2012) in the end-to-end mode, with the minimum score threshold L,-0.6,-0.9 and seed length 15. Reads were realigned with GATK to improve mapping around indels. Then, separate bam files were produced for individual MIPs and SNP-calling was carried out for each MIP using GATK UnifiedGenotyper with ploidy set to 4 to improve SNP-calling in duplicated genes and a minimum SNP quality of 20 Phred. At this stage per-MIP per-individual coverage was calculated.

Amplicons. Genotyping of *MHC-I* and *BRD2* was performed using the adjustable clustering method implemented in AmpliSAS (Sebastian et al. 2016). The genotyping thresholds used for each genus and amplicon type are given in Table S2. For genera *Batrachoseps* and *Eurycea MHC-I* exon 2 was amplified for each individual in three replicates and genotyping was based on per-amplicon frequencies of variants averaged over the replicates.

Diversity

As explained in Materials and Methods (main text) some APGs and nonAPGs were duplicated in some species. To maximize the use of the information contained in sequences and analyse all genes, including *MHC-I*, in a uniform framework, we utilized physically phased haplotypes obtained for short segments of studied genes (Fig. 3). Below we describe the entire procedure of obtaining segments, haplotypes within segments and per-segment diversity estimates.

For each MIP and each individual we reconstructed haplotypes with *microhaplot* R package, using only SNPs of quality ≥ 100. The coordinates of segments were obtained from bed file containing coordinates of all MIP targets on the reference sequence. We defined a segment as a continuous stretch of reference that was covered by a unique combination of MIPs

(one or more), such that each MIP fully covered the segment. Full sequences of all haplotypes within the segment were then easily obtained, as they were contained within single reads. At this stage, to increase the length of segments we filtered out MIPs with median coverage below 20 reads. Then for each haplotype in each individual we calculated its per amplicon frequency (PAF), i.e. the proportion of reads derived from a given MIP that represented the haplotype. Using segment coordinates we extracted for each individual all haplotypes in that segment derived from MIPs with coverage of at least 20 reads and PAF $\geq$ 0.1. Diversity was then calculated for all segments successfully resequenced in at least half the individuals of a given species. For nonpolymorphic MIPs, the minimum coverage of three was considered successful resequencing.

For each segment within a species phylogenetic α and γ diversities were calculated. BIONJ (Gascuel 1997) tree was constructed from all unique haplotypes using three measures of genetic distance: dS, i.e. the number of synonymous differences per synonymous site estimated using the method of Li (1993), raw protein divergence, or amino-acid *p*-distance and protein divergence expressed as the amino-acid Grantham distance (see Pierini and Lenz 2018). Although segments were short in most cases, comprising a single to dozens of codons, which caused generally poor resolution of the trees, this was not a major concern for our purposes, as phylogenies only served as a convenient way of summing the genetic distances between the haplotypes, not to interpret relationships.

References

Boyle EA, O'Roak BJ, Martin BK, Kumar A, Shendure J. 2014. MIPgen: optimized modeling and design of molecular inversion probes for targeted resequencing. *Bioinformatics* 30:2670-2672.

Elewa A, Wang H, Talavera-López C, Joven A, Brito G, Kumar A, Hameed LS, Penrad-Mobayed M, Yao Z, Zamani N. 2017. Reading and editing the *Pleurodeles waltl* genome reveals novel features of tetrapod regeneration. *Nat Comm* 8:1-9.

Fijarczyk A, Dudek K, Niedzicka M, Babik W. 2018. Balancing selection and introgression of newt immune-response genes. *Proc Roy Soc B* 285:20180819.

Gascuel O. 1997. BIONJ: an improved version of the NJ algorithm based on a simple model of sequence data. *Mol Biol Evol* 14:685-695.

Hiatt JB, Pritchard CC, Salipante SJ, O'Roak BJ, Shendure J. 2013. Single molecule molecular inversion probes for targeted, high-accuracy detection of low-frequency variation. *Genome Res* 23:843-854.

Kaufman J, Salomonsen J, Flajnik M. 1994. Evolutionary conservation of MHC class I and class II molecules - different yet the same. *Sem Immun* 6:411-424.

Langmead B, Salzberg SL. 2012. Fast gapped-read alignment with Bowtie 2. *Nat Meth* 9:357-359.

Li H, Durbin R. 2009. Fast and accurate short read alignment with Burrows–Wheeler transform. *Bioinformatics* 25:1754-1760.

Li W-H. 1993. Unbiased estimation of the rates of synonymous and nonsynonymous substitution. *J Mol Evol* 36:96-99.
McKenna A, Hanna M, Banks E, Sivachenko A, Cibulskis K, Kernytsky A, Garimella K, Altshuler D, Gabriel S, Daly M. 2010. The Genome Analysis Toolkit: a MapReduce framework for analyzing next-generation DNA sequencing data. *Genome Res* 20:1297-1303.
Niedzicka M, Fijarczyk A, Dudek K, Stuglik M, Babik W. 2016. Molecular Inversion Probes for targeted resequencing in non-model organisms. *Sci Rep* 6:24051.
Nowoshilow S, Schloissnig S, Fei J-F, Dahl A, Pang AW, Pippel M, Winkler S, Hastie AR, Young G, Roscito JG. 2018. The axolotl genome and the evolution of key tissue formation regulators. *Nature* 554:50-55.
Palomar G, Dudek K, Wielstra B, Jockusch EL, Vinkler M, Arntzen JW, Ficetola GF, Matsunami M, Waldman B, Těšický M. 2021. Molecular evolution of antigen-processing genes in salamanders: do they coevolve with MHC class I genes? *Genome Biol Evol* 13:evaa259.
Pedersen BS. 2014. Aligning sequence from molecular inversion probes. *bioRxiv*:007260.
Pierini F, Lenz TL. 2018. Divergent allele advantage at human MHC genes: signatures of past and ongoing selection. *Mol Biol Evol* 35:2145-2158.
Reche PA, Reinherz EL. 2003. Sequence variability analysis of human class I and class II MHC molecules: Functional and structural correlates of amino acid polymorphisms. *J Mol Biol* 331:623-641.
Sammut B, Du Pasquier L, Ducoroy P, Laurens V, Marcuz A, Tournefier A. 1999. Axolotl MHC architecture and polymorphism. *Eur J Immunol* 29:2897-2907.
Sebastian A, Herdegen M, Migalska M, Radwan J. 2016. amplisas: a web server for multilocus genotyping using next-generation amplicon sequencing data. *Mol Ecol Res* 16:498-510.
Zhang J, Kobert K, Flouri T, Stamatakis A. 2014. PEAR: a fast and accurate Illumina Paired-End reAd mergeR. *Bioinformatics* 30:614-620.

Figure S1. Sequence logos summarizing differences between MHC-I exon 2 and 3 protein sequences in salamanders.

The size of the letters reflects the relative frequency of a given amino-acid among all alleles detected in a genus. Key anchor positions in which particular amino-acids appear to be conserved in most classical MHC-I molecules (Kaufman et al. 1994; Sammut et al. 1999) are highlighted in blue; they were used to define the "conserved anchor" dataset. The peptide binding sites (excluding anchor residues) as defined in (Reche and Reinherz 2003) for human MHC-I are highlighted in yellow: darker - the positions common to HLA-A, B and C, lighter – the remaining positions.

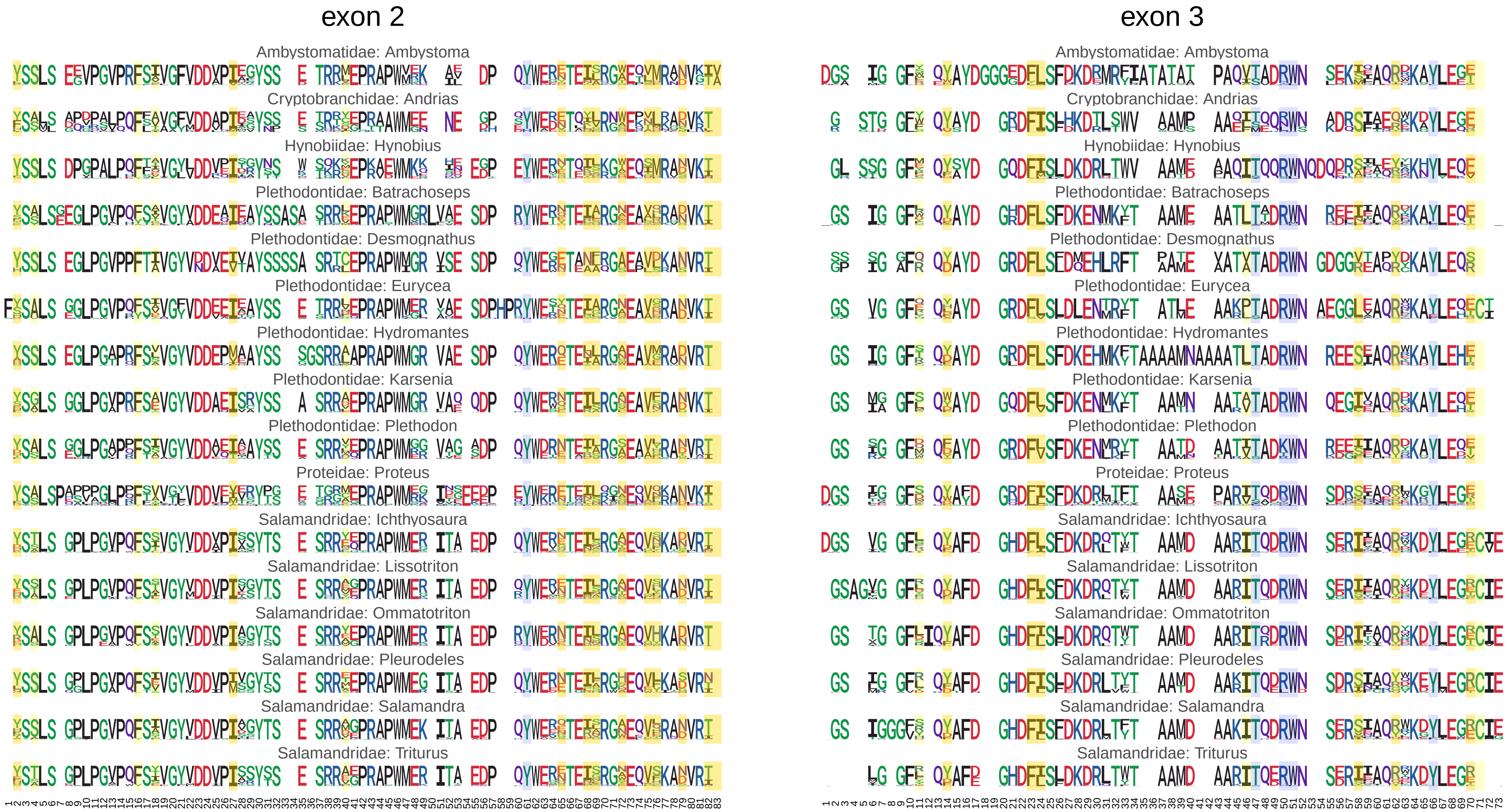
exon 2
exon 3
Ambystomatidae: Ambystoma
Cryptobranchidae: Andrias
Hynobiidae: Hynobius
Plethodontidae: Batrachoseps
Plethodontidae: Desmognathus
Plethodontidae: Eurycea
Plethodontidae: Hydromantes
Plethodontidae: Karsenia
Plethodontidae: Plethodon
Proteidae: Proteus
Salamandridae: Ichthyosaura
Salamandridae: Lissotriton
Salamandridae: Ommatotriton
Salamandridae: Pleurodeles
Salamandridae: Salamandra
Salamandridae: Triturus
